# Effect of Genetic Architecture and Partitioning of Training Population on GEBVs, SNP Effects and GWAS: A Simulation Study

**DOI:** 10.3390/genes17060670

**Published:** 2026-06-07

**Authors:** Gaurav Dutta, Hélène Wilmot, Elizabeth D. Schifano, Breno Fragomeni

**Affiliations:** 1 Department of Animal Science, University of Connecticut, Storrs, CT 06269, USA; gaurav.dutta@uconn.edu; 2 Department of Animal and Dairy Science, University of Georgia, Athens, GA 30602, USA; helene.wilmot@uga.edu; 3 Department of Statistics, University of Connecticut, Storrs, CT 06269, USA; elizabeth.schifano@uconn.edu; 4 Institute of Systems and Genomics, University of Connecticut, Storrs, CT 06269, USA

**Keywords:** genomic estimated breeding values, single nucleotide polymorphism effects, quantitative trait loci, accuracy, genomic prediction

## Abstract

Background/Objectives: Inconsistency of results in genome-wide association studies (GWAS) has been a challenge for animal breeders and geneticists. Understanding how different training subset configurations influence genomic estimated breeding values (GEBVs) and GWAS is essential for optimizing genomic evaluations. This study aimed to evaluate the impact of training population partitioning and QTL architecture on prediction accuracy, GEBV and SNP-effect correlations, and on the consistency of GWAS. Methods: A simulated population consisting of ten breeding generations was partitioned and evaluated on four training scenarios: animal ID, sex, generations, and generation correct.blocks. Moreover, four distinct genetic architectures were simulated, representing combinations of two QTL counts (100 and 1000) and two effect-size distributions (normal and gamma). Phenotypes were available for 10,000 individuals, which were genotyped for 50,000 SNP markers. Results: Across generation blocks, accuracy increased from earlier to more recent generations. GEBV correlations were consistently higher than SNP-effect correlations across scenarios. Adjacent generation blocks showed stronger correlations than distant blocks. Architectures with 1000 QTL yielded higher accuracy than 100 QTL architectures, while effect distribution had limited influence. Manhattan plots showed stable major QTL peaks across subsets. However, reduced peak magnitudes with more noise signals were observed in smaller training sets. Training population size and genetic distance strongly influenced genomic prediction performance. GEBVs were more stable than individual SNP-effect estimates across training configurations. Conclusions: These findings provide insights for interpreting why GWAS results fluctuate more than breeding values due to limited dimensionality of genomic information.

## 1. Introduction

Genomic selection enabled breeders to reduce the generation interval and increase selection accuracy in livestock populations, thereby accelerating the overall response to selection [1,2]. Additionally, the use of genomic information allows the identification of genomic regions associated with the phenotypes of interest. While the use of dense single-nucleotide polymorphism (SNP) marker panels has been useful for identifying selection candidates at younger ages, results from genome-wide association studies (GWAS) have been inconsistent across publications [3,4].

Identifying key quantitative trait loci (QTL) through GWAS is a critical step toward characterizing and understanding specific genetic variants significantly associated with economically important phenotypes in livestock. GWAS has evolved since the first implementation of genomic selection in livestock animals across multiple species [3,5,6,7]. These advances include the transition from single-marker regression to single-step GWAS frameworks, which leverage phenotypes from both genotyped and ungenotyped relatives to maximize statistical power [8]. Furthermore, the increasing accessibility of whole-genome sequence data [9] and multi-breed meta-analyses [10] has improved the resolution of association studies. Despite these advances, accurate estimation of SNP effects remains a fundamental challenge. Although modern and quite extensive reference populations often have individual records that outnumber the standard number of SNP markers, the problem remains for small breeds, hard-to-measure traits, or reduced population subsets. Nevertheless, a recurring issue in genomic evaluation is the large number of markers (p), small number of records (n) problem. Moreover, because the genotypes of causal mutations are seldom known, GWAS rely on the linkage disequilibrium (LD) between a marker and a QTL to capture the genetic signal. [11]. As allele frequencies and LD structures vary across datasets, the association signal fluctuates, yielding different effect estimates for the same locus. This explains why the same traits can yield inconsistent GWAS peaks when analyzed in different cohorts [12,13].

Building upon these concepts, genomic selection offers a framework to evaluate the total genetic merit of an individual. As proposed by Meuwissen et al. [14], if sufficient LD exists within a population, a dense panel of markers distributed across the genome can collectively capture the effects of the underlying QTLs. This theoretical framework enables the direct estimation of an individual’s genomic estimated breeding value (GEBV) [15,16]. The utility and success of genomic selection for breeders are primarily determined by its predictive accuracy. Accuracy depends on factors such as the number of QTL, marker density, heritability, and the size and relatedness of the reference population [17]. Differences in accuracy gain can arise from the composition of the training populations [18,19,20], the complexity of the genetic architecture of the trait [21,22] and the statistical approach used for the genomic prediction model [23,24]. While GWAS was not an initial target for genomic selection, its results can be incorporated into prediction to increase the accuracy of GEBVs [23,25,26]. GWAS data can be incorporated as SNP weights [25,27], selected variants from sequence data [28] and from filtering a subset of SNPs [29,30], or by exploiting biological variants [23]. However, the use of those outputs does not always improve accuracy [31] and may present practical challenges for commercial genetic evaluations. Moreover, the effect of genetic architecture and population structure on breeding values, SNP effects and GWAS results is sometimes difficult to evaluate in commercial livestock data.

One cost-effective way to test different breeding program scenarios is through simulation studies. Such studies may provide essential insights into trait dynamics, enabling evaluation of practical scenarios and methodologies to achieve defined breeding objectives. One advantage of this type of study is the ability to compare GEBVs and True Breeding Values (TBVs), since, in practice, the TBVs are unknown. Similarly, in simulations, the position and effect of causative variants are known, allowing the evaluation of genomic associations. Therefore, simulation studies allow the evaluation of factors such as population structure, training set composition, and heritability, among others, that affect both prediction accuracy and genomic associations.

In this context, the objective of this study was to evaluate the effect of different training population subsets on SNP effects estimates and GEBVs in a simulated population with different genetic architectures. Moreover, this study aimed to simultaneously evaluate the GEBV stability and SNP-effects reproducibility across different training subsets. By understanding how the different subsets affect the correlations between GEBVs, SNP effects, and prediction accuracies, we aimed to improve the understanding of the fluctuations in a GWAS and in predictions.

## 2. Materials and Methods

### 2.1. Data Simulation

**Figure 1:**
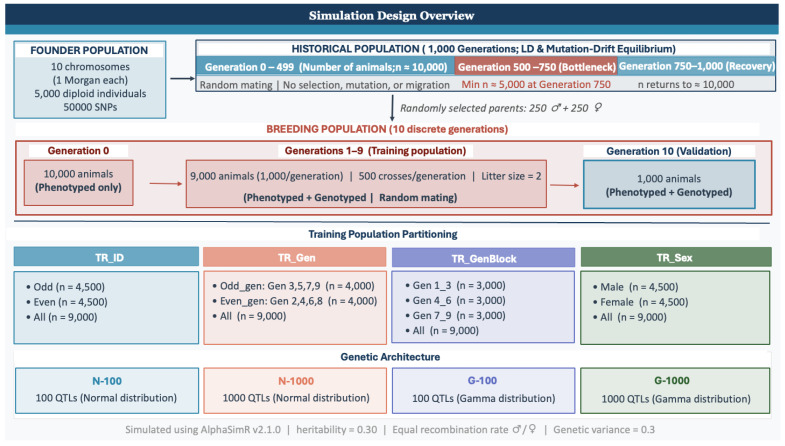
Schematic overview of the simulation design. The figure illustrates the progression from the founder population through the demographic bottleneck to the 10-generation breeding population, along with the training population partitioning strategies and genetic architectures evaluated. Abbreviations: TR_ID: partitioning by animal ID; TR_Gen: partitioning by generation; TR_GenBlock: partitioning by generation block; TR_Sex: partitioning by sex; N-100: normal distribution with 100 QTLs; N-1000: normal distribution with 1000 QTLs; G-100: gamma distribution with 100 QTLs; G-1000: gamma distribution with 1000 QTLs.

Genomic and phenotypic data were simulated with the R (Version 4.5.3) package AlphasimR v2.1.0 [32]. Figure 1 shows a schematic overview of how the simulation data were generated. Initially, a founder population was created by generating 10,000 haplotypes across 10 chromosomes, each with an equal length of 1 Morgan. Haplotypes were randomly sampled and paired to form 5000 diploid individuals, which served as the base population for subsequent simulations. For each chromosome, a genetic map was constructed to specify the positions of segregating sites, and haplotypes were generated by randomly assigning alleles (0 or 1) to the SNP loci. A single trait was simulated with a heritability of 0.30, and 100% of the genetic variance was explained by either 10 or 100 biallelic QTL per chromosome, resulting in 100 or 1000 total QTLs across the genome. To represent contrasting genetic architectures, QTL allelic effects were sampled from either a normal distribution with mean 0 and variance of 0.30, or a gamma distribution with a shape parameter of 0.4 internally scaled to ensure the true additive genetic variance equaled 0.30, resulting in four distinct simulation scenarios with the same heritability: N-1000 and G-1000 (normal and gamma distribution with 1000 QTLs, respectively) and N-100 and G-100 (normal and gamma distribution with 100 QTLs, respectively).

To create an initial LD and mutation-drift equilibrium, the founder population was expanded into a historical population simulated over 1000 non-overlapping generations, with the same recombination rate assumed for females and males. The mating was random, with no selection, mutation, or migration. From generation 0 to generation 499, each generation consisted of 10,000 individuals, generated by random mating among 2500 males and 2500 females. Specifically, n  males and n females were sampled each generation, and 4n crosses were generated with one progeny per cross, allowing parents to be reused across crosses within generation. To create linkage disequilibrium, a bottleneck was implemented between generations 500 and 750, with the population size gradually reduced from 10,000 individuals to a minimum of 5000 individuals at generation 750, achieved by proportionally reducing the number of crosses. Following the bottleneck, the population was gradually re-expanded between generations 750 and 1000, returning to 10,000 individuals per generation, where it remained until the end of the historical phase. A breeding population was subsequently created by randomly selecting 250 males and 250 females from the final generation of the historical population. Mating remained random in the breeding population, and litter size was fixed at two progeny per cross per generation, with 500 crosses across the randomly selected animals, resulting in 1000 offspring per generation with an effective population size of 500. The breeding population was simulated for 10 non-overlapping generations.

The phenotypes of the individuals were computed as the sum of the simulated TBV and a random error to achieve the desired heritability. Phenotypic data included the individual’s ID, sex, phenotypic value, TBV, and generation. Phenotypes were available for all individuals from the last generation of the founder population, hereby generation 0, with a total of 10,000 animals and the ten subsequent breeding generations, named generations 1–10. Genotypic data were generated only for individuals from the breeding population, yielding 10,000 genotyped individuals, each genotyped for 50,000 SNPs evenly distributed as markers, representing a 50k commercial SNP chip. Phenotypes from generation 10 were used for validation, while the training population had phenotypes from generations 1 to 9, consisting of 9000 animals. To ensure the robustness of the results and account for stochastic variation during the simulation of historical populations and QTL sampling, the entire simulation pipeline was repeated for five independent replicates. Each replicate used a unique seed while maintaining the previously described population parameters and genetic architectures.

The training population was later partitioned into different subsets, each representing a distinct training scenario used to estimate GEBVs and SNP effects. Initially, all animals from generations 1 to 9 composed the benchmark group, hereby termed All. In the first Scenario (TR_ID), animals were divided into two subsets based on their identification numbers, comprising odd- and even-numbered individuals, termed *Odd* and *Even*. Each group consisted of 4500 phenotypes and genotypes. In the second scenario (TR_Gen), animals were grouped by generation: generations 3, 5, 7, and 9 were used for the *Odd_gen* group, and generations 2, 4, 6, and 8 for the *Even_gen* group, each containing 4000 phenotypes and genotypes. Generation 1 was removed from this scenario to ensure a consistent number of records for a fair comparison. Additionally, generation blocks (TR_GenBlock) were defined and included data for non-overlapping groups of three generations: *Gen 1_3*, *Gen 4_6*, and *Gen 7_9*, consisting of generations 1, 2, and 3; 4, 5, and 6; and 7, 8, and 9, respectively, where each subset consisted of 3000 phenotypes and genotypes. Finally, in the fourth scenario (TR_Sex), animals were separated by sex into *male* and *female*, each comprising 4500 phenotypes and genotypes. Across all scenarios, Pearson correlations of GEBVs and SNP effects were computed between subsets, while prediction accuracy was assessed as the correlation between TBVs and GEBVs within each training configuration.

### 2.2. Model

The following model was used for single-trait analysis:

(1) y= 1μ + Za + e,

where ***y*** is the vector of simulated phenotypes; **1** is the vector of all ones; *µ* is the overall mean; **Z** is an incidence matrix that relates individuals to phenotypes; ***a*** is the vector of random additive genetic direct effects, and e is the vector of random residual effects. GEBVs were calculated using a single-step GBLUP methodology [33,34], which combines pedigree, phenotypes, and genomic information. Pedigree information of generations 0–10 was included. The assumptions for the random effects were:

(2) $$var\left[\begin{array}{c} a\\ e \end{array}\right]=\left[\begin{array}{cc} H{\sigma^{2}}_{a} & 0\\ 0 & I{\sigma^{2}}_{e} \end{array}\right],$$

where H is the combined pedigree–genomic relationship matrix. Random effects were defined as ***a****~N (0*, ***H*** *σ*^2^*_a_)*, where σ^2^*_a_* is the additive genetic variance and ***e*** *~ N (0*, ***I*** *σ*^2^*_e_)*, where **I** is the identity matrix and σ^2^*_e_* is the residual variance. Random effects variances were specified through simulation parameters, where σ^2^*_a_*= 0.3 and σ^2^*_e_* = 0.7. The inverse of this matrix **H** is required and can be directly obtained through the following form [35];

(3) $$H^{-1}=A^{-1}+\left[\begin{array}{cc} 0 & 0\\ 0 & G^{-1}-{A_{22}}^{-1} \end{array}\right],$$

where $G^{-1}$ is the inverse of the genomic relationship matrix [36], $A^{-1}\;$ is the inverse pedigree numerator relationship matrix, and $A_{22}$ is a numerator relationship matrix for genotyped animals.

### 2.3. Derivation of SNP Effects from Breeding Values

SNP effects (u) were estimated by back-solving the GEBV [8];

(4) $$u=\lambda M’G^{-1}a_{g},$$

where λ is a variance ratio (σ^2^_u/_${\sigma^{2}}_{a})$, σ^2^_u_ is the additive genetic variance captured by each SNP marker, ***M*** is a centered matrix of gene content, and **a*_g_*** is the vector of the animal effects. Finally, SNP effects were used to estimate the individual variance of each SNP effect [37]

(5) $$\sigma_{ui}^{2}=2p_{i}\left(1-p_{i}\right)u_{i}^{2},$$

where *p_i_* is the allele frequency of the reference allele used in SNP genotype coding and u*_i_* is the estimated additive effect of SNP *i*.

### 2.4. GWAS

Genome-wide association analyses were conducted to evaluate the genomic regions contributing to genetic variance and to compare estimated SNP effects with the underlying simulated QTL architecture. Because each chromosome was simulated as 1 Morgan with 10,000 SNPs, adjacent markers were spaced at 0.0001 Morgan. To reduce noise from individual back-solved SNP effects while retaining local genomic resolution, SNP effects were summarized in sliding windows of 20 adjacent markers, corresponding to 0.002 Morgan (0.2 cM) per window. This window size was chosen to aggregate clusters of neighboring SNPs likely to capture the same local LD signal from an underlying QTL, thereby improving visualization of regional association peaks. The variance explained by each window was calculated as the sum of the variances of the SNPs within that window. For validation of the GWAS peaks, the true genetic variance explained by each simulated QTL was calculated using Falconer’s quantitative genetics formulation [38]:

(6) $$\sigma_{QTL}^{2}=2p_{i}\left(1-p_{i}\right)\alpha^{2},$$

where 1 – *p_i_* is the frequency of the alternative allele and α is the additive simulated QTL effect. This formulation provides the theoretical additive genetic variance contributed by each locus under Hardy–Weinberg equilibrium. The calculated QTL variances were used to determine the proportion of total QTL variance attributable to each locus and were represented on Manhattan plots to compare simulated genetic architecture with the estimated SNP effects in various subset scenarios. A visual inspection of the Manhattan plots was conducted to evaluate the detection power of the major simulated peaks.

To quantify GWAS reproducibility across training population subsets, SNP windows were ranked by the proportion of genetic variance explained. For this comparison, overlapping windows were removed prior to selection, retaining only the highest-ranked non-overlapping window per genomic region. For each genetic architecture and replicate, the top 10, 100, and 200 non-overlapping SNP windows were identified across subsets. Pairwise reproducibility was assessed by counting overlapping windows between subset pairs. Results were summarized across five replicates as mean ± standard error. Additionally, given that true causal QTL positions were known from the simulation, SNP windows were further classified as true-positive (TP) or false-positive (FP) based on their overlap with simulated QTL regions, for 100 QTL genetic architectures. The same procedure was implemented for the 1000 QTL architecture, but only using the top 100 QTL, since most simulated variants in this scenario had small effects and covered a large portion of the genome, as including all QTL would inflate the TP rate. For each replicate and subset, windows were ranked by proportion of variance explained and evaluated against architecture-specific thresholds: 0.20 for N-100, 0.30 for G-100, and 0.10 for N-1000 and G-1000. A window was classified as TP if it exceeded the threshold and overlapped at least one simulated QTL region, and FP if it exceeded the threshold but did not overlap any simulated QTL region. The total number of windows above the threshold, TP windows, and FP windows was recorded per replicate and summarized across five replicates. This approach provided a quantitative assessment of GWAS signal reliability, distinguishing reproducible high-ranking genomic windows capturing true causal regions from those that did not correspond to known QTL positions.

### 2.5. Correlation and Validation

The prediction accuracy was calculated as the Pearson correlation coefficient between the simulated TBV and the GEBV for each scenario for the 1000 validation animals in generation 10:

(7) Accuracy=cor(TBV,GEBV),

Those animals did not have their phenotypes included in the analysis, which mimicked selection for young breeding candidates. Additionally, correlations between GEBVs of 10th-generation animals across different training subsets were calculated to assess the consistency of genomic predictions. Pairwise correlations of SNP effects estimated from the same subsets were also computed to evaluate the stability of marker-effect estimates across training scenarios. Together, these correlations provided a measure of the stability of genomic predictions over generations. Tukey’s honest significant difference (HSD) test was employed to evaluate pairwise differences in genomic prediction accuracy, GEBV correlations, and SNP-effect correlations across replicates, both among genetic architectures and across training subset comparisons within each architecture [39].

### 2.6. Software

All genomic evaluations and GWAS were performed using the BLUPF90 family of programs (v1.1.0). All graphics, including Manhattan plots, correlations, and accuracies, were calculated using R studio (v4.5.3) and ggplot2 package (v4.0.2) [40].

## 3. Results

The standard errors for prediction accuracy among five replicates were less than 0.01; thus, results are presented as replicate means. The following sections present results from genomic prediction analysis and interpretation of GWAS using different simulated training subsets based on the four scenarios: TR_ID, TR_Gen, TR_GenBlock, and TR_Sex and four genetic architectures based on distribution and number of QTLs (N-1000, G-1000, N-100, and G-100). Subset comparisons within each simulated scenario were evaluated based on GEBV accuracy, pairwise GEBV correlations, pairwise SNP-effect correlations, and visual inspection of Manhattan plots complemented by quantitative reproducibility validation. Detailed results including means, standard errors, and adjusted *p*-values are summarized in Supplementary Tables S1–S5.

### 3.1. Accuracy of GEBVs

**Figure 2:**
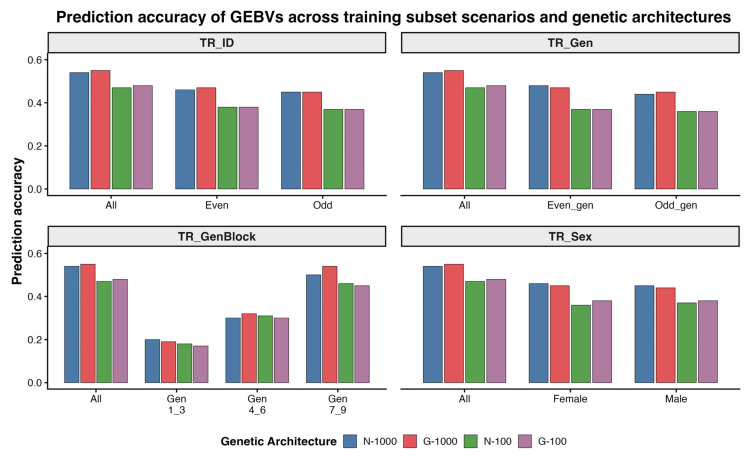
Prediction accuracy of GEBVs across four training population subsets and four simulated genetic architectures. Training subsets were partitioned by animal identification numbers (TR_ID: *All*, *Odd*, *Even*), alternating generations (TR_Gen: *All*, *Odd_gen*, *Even_gen*), generation blocks (TR_GenBlock: *Gen 1_3*, *Gen 4_6*, *Gen 7_9*), and sex (TR_Sex: *Male*, *Female*). Genetic architectures (*N-1000*, *G-1000*, *N-100*, *G-100*) were denoted by QTL effect distribution (N: Normal; G: Gamma) and total QTL density (100 or 1000 causative loci).

Figure 2 presents the prediction accuracies of GEBVs for training subsets across different training populations of the subset scenarios and genetic architectures. Across all scenarios, the highest accuracies were obtained with the complete training datasets. Accuracy tended to be higher in the 1000 QTL scenarios and in those with more data in the training set. Accuracies were comparable when the number of individuals was the same for O*dd* and E*ven* identification numbers, *Male* and *Female*, and odd and even generation numbers. In the scenario TR_Genblock, prediction accuracy increased as the training dataset became closer to the validation dataset. The normal and gamma distributions had similar accuracies in 1000 and 100-QTL scenarios. 

### 3.2. Correlations Between GEBVs

**Figure 3:**
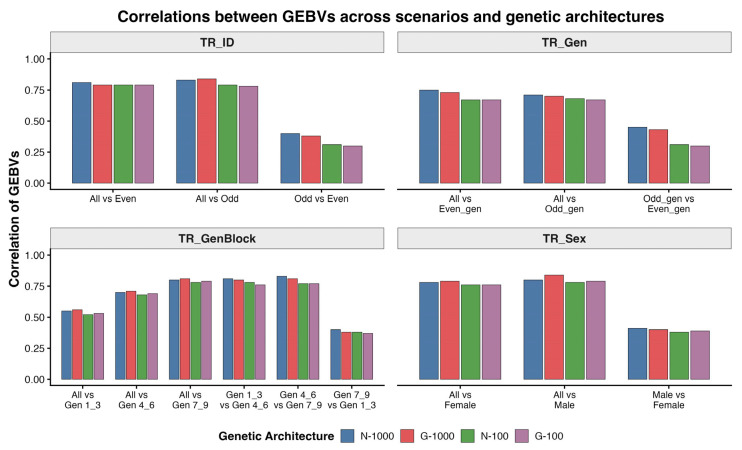
Correlations between genomic estimated breeding values (GEBVs) across four training population subsets and four simulated genetic architectures. Training subsets were partitioned by animal identification numbers (TR_ID: *All*, *Odd*, *Even*), alternating generations (TR_Gen: *All*, *Odd_gen*, *Even_gen*), generation blocks (TR_GenBlock: *Gen 1_3*, *Gen 4_6*, *Gen 7_9*), and sex (TR_Sex: *Male*, *Female*). Genetic architectures (*N-1000*, *G-1000*, *N-100*, *G-100*) were denoted by QTL effect distribution (N: Normal; G: Gamma) and total QTL density (100 or 1000 causative loci).

Pearson correlations between GEBVs estimated from different training subsets are shown in Figure 3. Correlations between the complete and reduced datasets were consistently higher than correlations between the reduced subsets. Moreover, correlations were consistent within and across genetic architectures. However, in the 100 QTL scenarios compared to 1000 QTLs, the correlations were slightly lower when calculated between reduced subsets. In TR_GenBlock, correlations between adjacent generation blocks were similar and higher than those between more distant generation blocks.

### 3.3. Correlations Between SNP Effects

**Figure 4:**
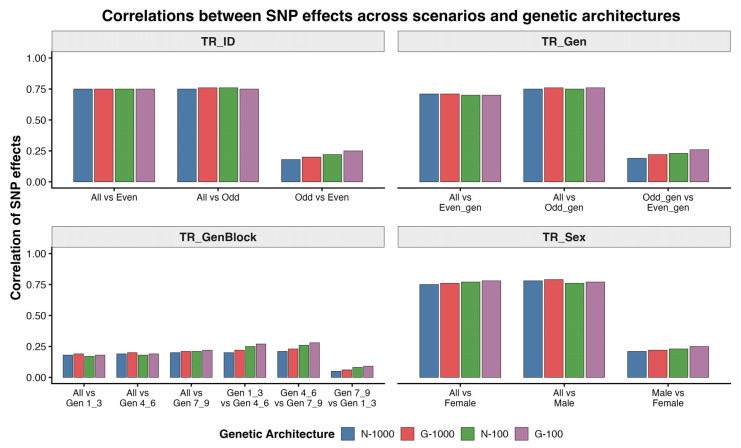
Correlations between SNP effects estimated across four training population subsets and four simulated genetic architectures. Training subsets were partitioned by animal identification numbers (TR_ID: *All*, *Odd*, *Even*), alternating generations (TR_Gen*: All*, *Odd_gen*, *Even_gen*), generation blocks (TR_GenBlock*: Gen 1_3*, *Gen 4_6*, *Gen 7_9)*, and sex (TR_Sex*: Male*, *Female*). Genetic architectures (*N-1000*, *G-1000*, *N-100*, *G-100*) were denoted by QTL effect distribution (N: Normal; G: Gamma) and total QTL density (100 or 1000 causative loci).

Figure 4 illustrates Pearson correlations between SNP effects estimated from different training subsets. In scenarios TR_ID, TR_Gen and TR_Sex, SNP-effect correlations between the complete and reduced training subsets were higher than those within the subsets. The SNP effect correlations were constant within genetic architectures, with some minor differences observed in the comparisons within subsets. In scenario TR_GenBlock, SNP effect correlations were lower than in other scenarios. These correlations were higher between adjacent blocks and markedly lower between more distant blocks.

### 3.4. Manhattan Plots

**Figure 5:**
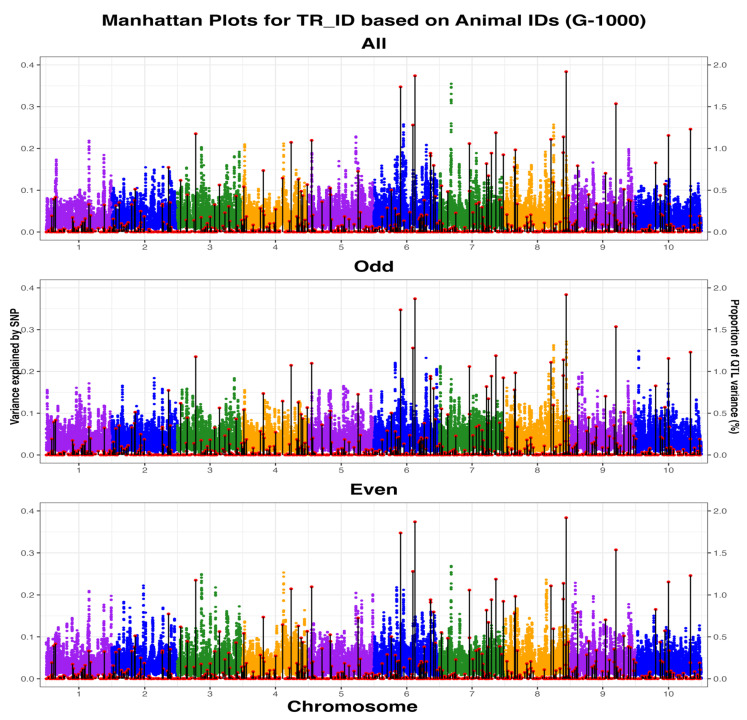
Manhattan plots for the G-1000 genetic architecture across TR_ID subsets *(All*, *Odd*, *and Even)* based on animal identification numbers. Points represent the proportion of genetic variance explained by individual SNPs across the 10 simulated chromosomes. Each point represents an individual SNP, with alternating colors used to distinguish ten chromosomes across the genome. Red markers indicate the true simulated QTL effects, scaled to the secondary *y*-axis as a proportion of QTL variance explained.

**Figure 6:**
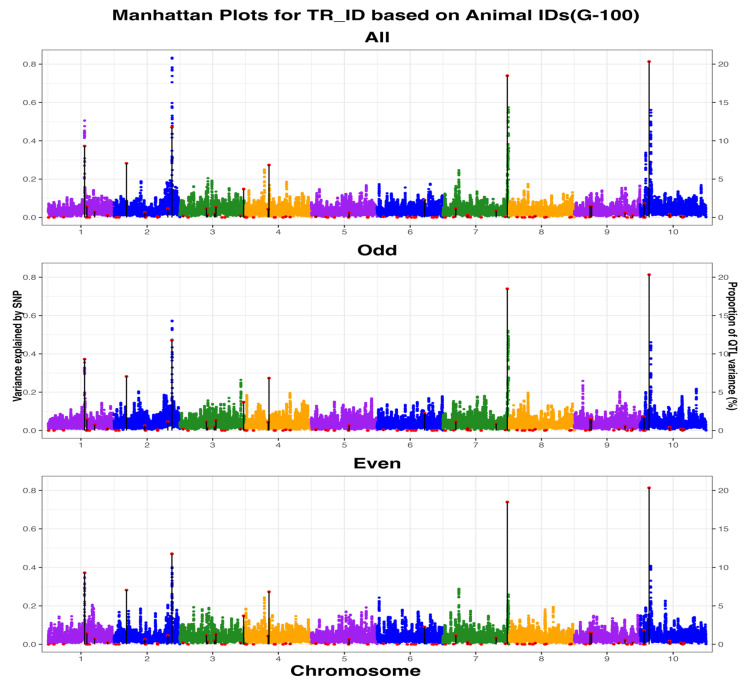
Manhattan plots for the G-100 genetic architecture across TR_ID subsets (*All*, *Odd*, *and Even)* based on animal identification numbers. Points represent the proportion of genetic variance explained by individual SNPs across the 10 simulated chromosomes. Each point represents an individual SNP, with alternating colors used to distinguish ten chromosomes across the genome. Red markers indicate the true simulated QTL effects, scaled to the secondary *y*-axis as the proportion of QTL variance explained.

Manhattan plots illustrating SNP variance explained by 20 adjacent SNPs across the 10 chromosomes for the subsets based on scenario TR_ID: *All, Odd*, and *Even* IDs are presented in Figure 5 for the G-1000 architecture and in Figure 6 for the G-100 architecture. Under the G-1000 architecture, prominent peaks were observed on multiple chromosomes in the complete training population. In the reduced subsets, peak heights were lower than in the complete dataset, and new peaks were observed. The relative positions of the highest peaks were inconsistent between subsets. In the G-100 architecture, most major simulated peaks were detected both in the complete and reduced subsets, with some false-positive peaks observed in the reduced datasets.

**Figure 7:**
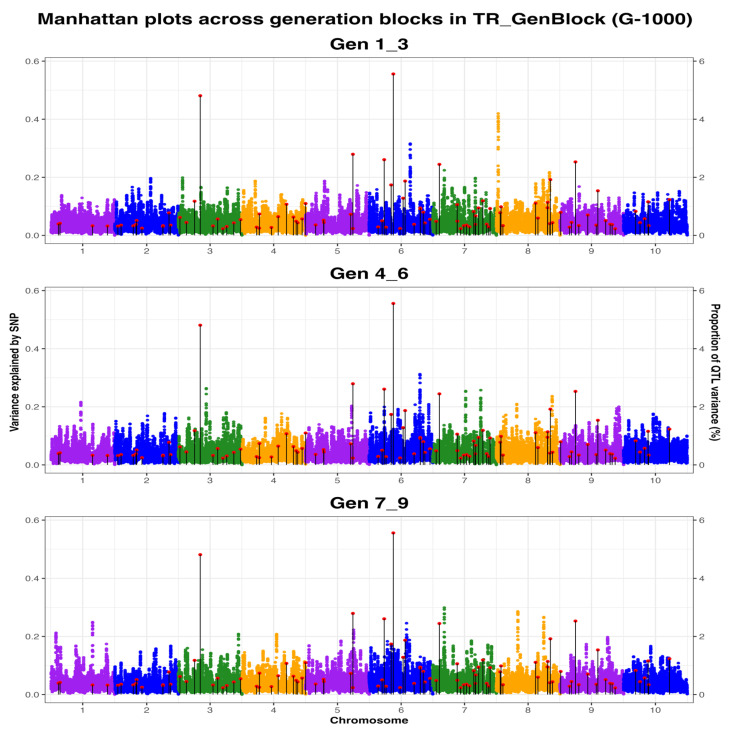
Manhattan plots for the G-1000 genetic architecture across three generation blocks (*Gen 1_3*, *Gen 4_6*, *and Gen 7_9)* under the TR_GenBlock scenario. The *x*-axis indicates chromosome position, the left *y*-axis shows the variance explained by SNP, and the right *y*-axis shows the corresponding proportion QTL variance (%). Each point represents an individual SNP, with alternating colors used to distinguish chromosomes across the genome. Red markers indicate the true simulated QTL effects, scaled to the secondary *y*-axis as the proportion of QTL variance explained.

**Figure 8:**
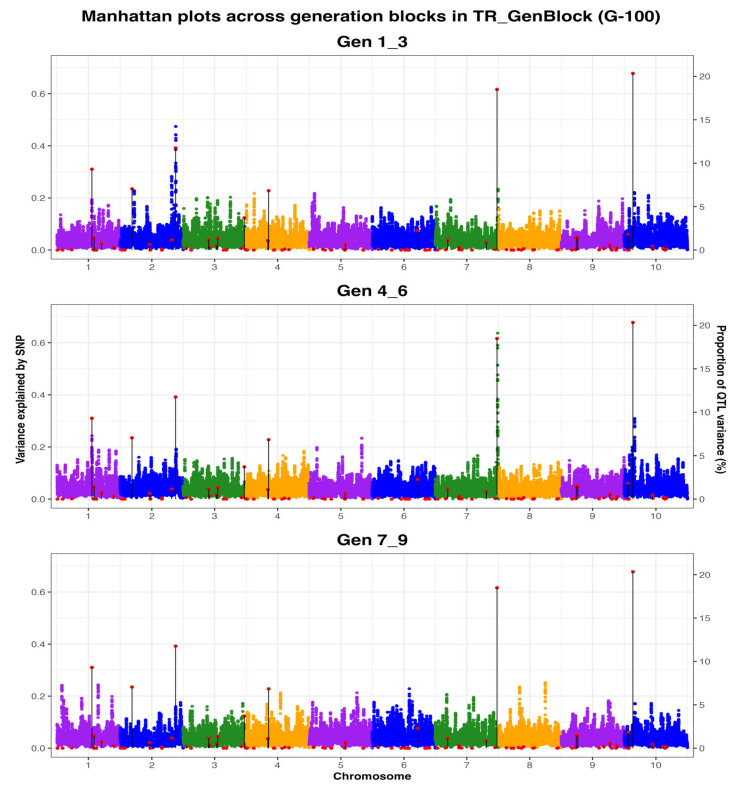
Manhattan plots for the G-100 genetic architecture across three generation blocks (Gen 1_3, Gen 4_6, and Gen 7_9) under the TR_GenBlock scenario. The *x*-axis indicates chromosome position, the left *y*-axis shows the variance explained by SNP, and the right *y*-axis shows the corresponding proportion QTL variance (%). Each point represents an individual SNP, with alternating colors used to distinguish ten chromosomes across the genome. Red markers indicate the true simulated QTL effects, scaled to the secondary *y*-axis as the proportion of QTL variance explained.

Manhattan plots were generated for the three training-generation blocks in TR_GenBlock scenario with the G-1000 (Figure 7) and G-100 (Figure 8) genetic architecture. In the G-1000 architecture, across all three generation blocks, a polygenic genomic architecture was observed, but the peaks varied across subsets. In each generation block, most SNP effects were observed at low variance-explained values, while a smaller number formed distinct peaks. The highest peak was located near chromosome 8 in *Gen 1_3*, whereas in *Gen 4_6* and *Gen 7_9* the tallest peaks were observed near chromosome 6. In *Gen 7_9*, SNP effect peaks appeared slightly sharper and more concentrated around major QTL positions. In *Gen 4_6*, peak heights were moderately reduced compared to *Gen 7_9*. In *Gen 1_3*, SNP signals were more dispersed, and peak magnitudes were generally lower relative to the more recent generation blocks. In the G-100 architecture, the highest peaks varied more often; near chromosome 2 in *Gen 1_3* and chromosome 7 in *Gen 4_6* and were inconsistent in *Gen 7_9*.

For the G-100 architecture, a distinct genomic profile was observed, characterized by sharp, high-magnitude peaks that closely aligned with the primary simulated QTL positions. In contrast to the more polygenic G-1000 scenario, the locations of the major signals remained highly consistent across all three generation blocks, although their relative intensities and resolution varied. The most prominent peaks were consistently identified on chromosomes 2, 7 and 9 across the entire TR_GenBlock configuration. In *Gen 1_3*, while the primary signals were evident, they were accompanied by higher background noise and more dispersed signals across other chromosomes, such as chromosome 2. As the training subsets approached the validation generation, the resolution of these signals improved; in *Gen 4_6* and *Gen 7_9*, the peaks on chromosomes 7 and 9 became significantly more concentrated and reached higher variance-explained values. By *Gen 7_9*, the Manhattan plots exhibited the highest signal-to-noise ratio, with SNP effects almost exclusively clustered around the major QTL markers, reflecting a precise capture of the underlying large-effect loci as generational distance was minimized. Manhattan plots for scenarios TR_Gen and TR_Sex were not presented as they were redundant and did not provide any additional distinct patterns. The Manhattan plots with other scenarios and genetic architectures are presented in Supplementary Figures S1–S12.

Pairwise overlap of top-ranked non-overlapping SNP windows across TR_ID subsets is presented in Table 1. Across all genetic architectures, shared window counts were consistently lower for *Odd* vs. *Even* comparisons relative to *All* vs. *Odd* and *All* vs. *Even* comparisons. The proportion of shared windows relative to the total selected remained stable across all subset comparisons and genetic architectures. Architectures with a higher number of QTLs (N-1000 and G-1000) showed greater overlap compared to those with fewer QTLs (N-100 and G-100) across all subset comparisons.

QTL-based classification of top SNP windows across TR_ID subsets is summarized in Table 2. Across all genetic architectures, the *All* subset consistently yielded the highest number of TP windows and the lowest number of FP windows relative to the *Odd* and *Even* subsets. FP window counts were notably higher in the *Odd* and *Even* subsets compared to the *All* subset across all architectures. Architectures with 100 QTLs (N-100 and G-100) showed fewer total windows above the threshold compared to 1000 QTL architectures (N-1000 and G-1000), consistent with the more concentrated genetic variance in simpler architectures.

## 4. Discussion

This section discusses the prediction accuracy, correlations between GEBVs and SNP effects, and Manhattan plots across four simulation scenarios and genetic architectures in different training subsets. Reducing sample size resulted in reduced prediction accuracy and lower correlations between GEBVs and SNP effects. The correlations of SNP effects were more sensitive to training subsetting than GEBV correlations. GWAS signal detection was also assessed by visual inspection of Manhattan plots, which were sensitive to subsetting of the training population.

### 4.1. Training Population Size and Partitioning

The accuracy of genomic predictions depends on the number of animals in the training population, heritability, the number of independent chromosome segments, and the number of QTLs affecting the trait [17,18,19,21,22]. Accuracy can plateau once the training population reaches an optimal size, capturing most genetic variation [41,42]. In the present study, across all the scenarios, accuracy increased with the size of the training population. This pattern was expected from genomic predictions where accuracy and GEBV correlations increased with sample size. Daetwyler et al. [43] derived an approximation for accuracy showing that acc = √(Nh^2^/(Nh^2^ + M)), where *N* is training size and M = min(M_e_,n_QTL_), where n_QTL_ is the number of causative loci. Within a population, M_e_ represents the number of independently segregating chromosome segments. Under this framework, for more polygenic traits where n_QTL_ > M_e_, accuracy is limited by the ability to capture chromosome segments. Conversely, for simpler architectures where n_QTL_ < M_e_, the accuracy is primarily governed by the recovery of specific QTL effects. The effects of each segment are estimated indirectly when predicting genomic breeding values for individuals within a given population [44,45].

Random animal IDs, generation, and sex-based subsets introduce sampling variability and incomplete representation of population structure, leading to unstable estimates of marker effects and lower accuracy. In these random subsets, the moderate loss in accuracy was likely a function of the reduced sample size rather than a structural breakdown of the population, since sex and animal ID were not simulation parameters that would influence the population structure. Conversely, partitioning by generation systematically increases the genetic distance between training and validation cohorts. In a study by Muir [46], LD between causal QTLs and marker SNPs progressively decays over successive generations due to continuous recombination. Therefore, generation-based subsets weaken LD persistence and erode the realized genomic relationships between the cohorts. Complete datasets maintain higher accuracy by encompassing diverse haplotypes and minimizing these losses, while reduced subsets amplify the risk of statistical overfitting, where models such as ssGBLUP begin to capture subset-specific sampling noise rather than true additive genetic variance. Habier et al. [11] demonstrated that genomic prediction captures both LD between markers and QTL and realized relationships among individuals. Therefore, when subsets are analyzed separately, part of the relationship information exploited by genomic prediction is lost.

While GEBV prediction accuracies experienced moderate declines when the data were partitioned, the estimated SNP effects exhibited severe fluctuations. To understand this instability, it is critical to evaluate the role of M_e_ within the context of training size. When training size is reduced through subsetting, the ratio of genotyped markers to sampled animals becomes even more extreme. Because thousands of markers are condensed into a limited number of M_e_ segments, the markers are highly multicollinear. Such multicollinearity inflates the variance of each SNP’s predictor, causing the values to fluctuate drastically when a different subset of animals is used to compute the marker effect. This problem is compounded since reduced datasets lack the degrees of freedom required to independently resolve the true effects of these highly correlated SNPs. Consequently, while the sum of these effects, i.e., GEBV, remains robustly buffered across subsets, the exact weights assigned to individual SNPs fluctuate randomly depending on the specific individuals randomly sampled and the exact haplotype frequencies captured in that specific partition. Additionally, a small training size may result in underrepresented haplotypes throughout the population, further accentuating this problem. Therefore, as training size decreases, marker effect estimators become highly unstable due to inflated sampling variance and the stricter structural limitations imposed by M_e_ [47].

### 4.2. QTL Number and Distribution

The multicollinearity of SNPs interacts strongly with the underlying genetic architecture. In our study, the stability of the GWAS peaks across subsets was more pronounced in the 100 QTL architecture compared to the more polygenic architecture (1000 QTL). When a trait is controlled by fewer loci, the genetic variance is concentrated into stronger, distinct signals. These major-effect loci generate a mapping signal robust enough to consistently overcome the background sampling variance, anchoring the model’s SNP weights regardless of the data subset. Conversely, in the 1000 QTL scenario, the variance is dispersed so thinly across the genome that the true signals become indistinguishable from the noise of multicollinear markers. In this highly polygenic state, the model struggles to pinpoint major regions, exacerbating the fluctuation of effect estimates and resulting in highly unstable, subset-dependent Manhattan plots.

While the 100 QTL architecture provided greater stability for GWAS mapping, it yielded lower overall prediction accuracies than the 1000 QTL scenario. This outcome contrasts with theoretical expectations; as Daetwyler et al. [43] established, oligogenic traits should achieve higher prediction accuracies at smaller sample sizes because there are fewer effects to estimate. The divergence between our empirical results and this theoretical baseline is driven by the statistical priors of the predictive model. Methods like GBLUP or ssGBLUP assume an infinitesimal genetic architecture, distributing variance homogeneously across all markers. Consequently, the model performed optimally in the 1000 QTL scenario, which satisfied this assumption. In contrast, applying uniform shrinkage to the 100 QTL scenario penalized the true large-effect loci and assigned noise to non-causal regions, ultimately reducing the prediction accuracy for the oligogenic trait. As demonstrated by Morgante et al. [48], the prediction accuracy of quantitative traits is highly dependent on the alignment between the true genetic architecture and the assumptions of the chosen statistical model.

### 4.3. Generational Distance and LD Persistence

Empirical studies in livestock show decreasing predictive ability with increasing genetic distance across distinct populations or generations [49]. In TR_GenBlock, different generation blocks revealed clear temporal effects: prediction accuracy increased from early to recent generations based on their distance to the validation animals. This pattern reflects the decay of LD phase consistency and genomic relationships across generations. As demonstrated by Habier et al. [11,50], the predictive ability of markers decays over generations because continuous recombination events break the historical LD between markers and causal loci. On the other hand, when LD is stronger, prediction accuracy can be maintained, even in multi-breed and multi-generation scenarios [51]. However, when relatedness weakens, accuracy declines, especially for SNP-level effects. Moreover, Muir [46] reported that LD between QTL and SNP will decrease over generations, causing a decrease in the reliability of genomic prediction. The degree of relatedness between training and validation sets strongly dictates prediction accuracy. While close relationships maximize accuracy by leveraging shared long haplotype blocks [17], distant relationships degrade predictive performance by inflating noise and bias within the genomic relationship matrix [52]. In our study, the higher GEBV correlations between adjacent blocks compared to distant blocks are consistent with progressive erosion of genomic relationships over time [11,53].

SNP-effect correlations declined more sharply than GEBV correlations across generations. This contrast occurs because genomic breeding values are aggregates across thousands of loci, making them highly robust to the estimation errors of individual markers. Estimated SNP effects are strictly bound to the LD phase of the training subset. Over successive generations, continuous recombination reshuffles historical haplotypes, shifting the LD phase between markers and the true causal quantitative trait loci (QTL). Increasing genetic distance, genetic interactions, and substitution effects diminish the correlation of SNP effects across generations [54]. Richter et al. [55] investigated the changes in SNP effects in a population under genomic selection, where changes among correlations between SNP effects from the start of genomic selection to the last interval show that SNP effects changed over time. Therefore, while prediction models can maintain high accuracy of GEBVs by capturing broad familial relationships, the underlying marker effects behave as transient, localized approximations that cannot be reliably transferred or correlated across distant generations.

### 4.4. GEBVs and SNP Effects Stability

Across all scenarios, GEBV correlations between training subsets were consistently higher than SNP effect correlations, a pattern that held across all four genetic architectures and partitioning strategies. In most scenarios, correlations of GEBVs between the full and reduced subsets indicated that structurally similar subsets preserve prediction power, i.e., higher correlations between reduced and full subsets. SNP effect correlations had a similar pattern with overall lower correlations. However, between reduced subsets, GEBV and SNP effect correlations decreased, with a substantial drop in the latter. SNP effects are estimated with considerable uncertainty due to multicollinearity among markers and SNP chip density [14]. SNPs close to each other on a chromosome tend to be inherited together. This creates redundant information, making it difficult for statistical models to differentiate the individual effect of each marker. This may lead to noisy and unreliable estimates due to small changes in training data. With fewer phenotypic observations to inform the model, the ability to decouple markers in high LD was diminished. This intensified the impact of multicollinearity, leading to the observed instability, i.e., lower correlations in individual SNP effect estimates. While ssGBLUP avoids the direct multicollinearity issues inherent in marker-regression models by fitting animal effects, instability arises during the back-transformation process. Because markers in high LD contribute redundant information to the G matrix, the mathematical derivation of SNP effects from breeding values cannot uniquely partition genetic variance among highly correlated markers. This results in a fluctuation in individual SNP effect solutions, particularly when the training population size is reduced.

Furthermore, genomic prediction functions independently of precise causal loci recovery. Its primary utility lies in its ability to exploit realized genomic relationships to account for additive genetic variance, regardless of whether the underlying causative mutations are identified. This allows prediction accuracy to persist even when estimated SNP effects themselves are noisy or inconsistently allocated across markers [11,56]. GEBVs are robust to this noise because they represent the cumulative genetic signal. Therefore, when aggregated into genomic breeding values, estimation errors of markers partially cancel out, resulting in more stable predictions of GEBVs [57]. A possible reason for that is that the genomic relationship matrix is non-positive-definite for most livestock populations [36]. Consequently, solving the mixed model equations in genomic selection requires approximating its inverse, often achieved by blending, which can be obtained by adding a small percentage of the pedigree numerator relationship matrix to the genomic relationship matrix [36] or by genomic recursions [58]; thus, the GRM may have infinitely many approximate inverses. While the use of approximate inverses does not affect the prediction of breeding values due to the reduced dimensionality of the genomic information [59,60], it may also mean that there are infinite combinations of SNP effects that yield the same GEBV due to multicollinearity, suggesting that SNP effects may behave as non-estimable functions due to their potential fluctuations and high variability.

In the generation blocks scenario, TR_GenBlock, the correlations between GEBVs demonstrated the impact of genetic distance on prediction stability. While correlations were higher when comparing adjacent generation blocks, a sharp decline was observed when comparing distant, non-overlapping blocks. Additionally, a major reduction in accuracy for reduced subsets was observed in comparison with the other scenarios. This degradation is primarily driven by the decay of realized genomic relationships and the recombination-driven breakdown of LD phases between the training and validation sets over time. Notably, accuracy estimators, such as those described by Daetwyler et al. [43] may not fully capture these losses, as they often assume a stable relationship structure and do not account for the rapid erosion of genetic connectivity across non-overlapping generations.

### 4.5. GWAS Stability and Interpretation

The Manhattan plots complement the SNP effect correlation results by visualizing how estimated marker effects are distributed across the genome for each training subset. Since the simulation assumed no selection, differences in peak patterns across subsets reflect the influence of training population composition on effect estimation rather than changes in the underlying QTL architecture that could be observed in real data due to selection. Such fluctuations in GWAS peaks across population subsamples were previously observed in real data [61,62], but since they could have multiple causes such as selection [63], population structure [29,64], or genotype-by-environment interactions [65,66], the present simulation removes these confounding factors, allowing the fluctuations to be attributed solely to training subset composition.

Most major genomic regions corresponding to simulated QTL positions were consistently recovered across the complete and reduced subsets under both normal and gamma distributions with 100 simulated QTLs, even though false positives and negatives were observed. This is expected because large-effect QTL explain a larger share of additive genetic variance, so even with reductions in training population size, it is still possible to capture SNP associations [47,67]. In contrast, SNPs tagging smaller-effect QTL showed more variable proportions of variance explained across estimated effects across subsets, consistent with the lower signal-to-noise ratio inherent to polygenic architectures under reduced reference sizes, which was previously described in a poultry population when SNP associations were inconsistent across generations [62]. In real data, such changes observed across generations can be explained by selection that affects allele frequency and may affect LD between the marker and the causative variant [68]. However, in the present study, selection was not simulated, and the fluctuations cannot be explained by changes in LD or minor allele frequency across random subsets. A possible explanation is the limited number of independent chromosome segments in this population, which reflects real livestock data [69]. Such a limitation increases the number of SNPs per chromosome segment, leading to multicollinearity and higher variance inflation factors for the SNP effect estimators [70]. The quantitative assessment of SNP-window overlap and QTL-based window classification provided additional support for the observed instability of marker effect estimates across training subsets. Pairwise overlap of top-ranked non-overlapping SNP windows was consistently lower for reduced data set comparisons relative to other comparisons across all genetic architectures, with the proportion of shared windows remaining low regardless of window size. The complete subset consistently yielded the highest true-positive and lowest false-positive window counts across all architectures, whereas polygenic architectures showed higher false-positive counts relative to simpler architectures. These findings collectively confirm that training population composition and genetic architecture substantially influence the reproducibility of GWAS signals, independent of changes in the underlying QTL positions or true effect sizes.

In Scenario TR_GenBlock, the Manhattan plots revealed distinct variations in the distribution of the variance explained by SNPs across the non-overlapping generation blocks. While the underlying simulated genetic architecture, specifically, the QTL positions and true effect sizes, remained strictly constant, the estimated marker effects varied considerably from one subset to another. These variations highlight the instability of marker effect estimation when sampling from populations with differing localized LD patterns. Over successive generations, continuous recombination breaks down and reshuffles historical haplotypes. Consequently, the LD between marker SNPs and the fixed causal loci shifts randomly depending on the subset. Because the statistical mapping between markers and QTLs is highly sensitive to these subset-specific haplotype structures, the model assigns different marker weights to capture the same underlying genetic signal. This instability is consistent with the near-zero SNP effect correlations observed between the most distant generation block subsets, underscoring that without the stabilizing force of selection, the distribution of estimated effects fluctuates randomly as the training population’s LD structure changes over time [11], in addition to instabilities observed due to the limited dimensionality of genomic information.

### 4.6. Practical Implications

Genomic prediction accuracy is primarily determined by training population size, genetic relatedness between training and validation sets, persistence of LD phase across generations and underlying genetic architectures [17]. Our results indicate that GEBVs remain stable and preserve their prediction power even in reduced data sets. However, the subsets must be representative of the population so that M_e_ effects can be adequately captured [71]. Consistent phenotypic collection is necessary to achieve higher accuracies [72]. Contrary to GEBV predictions, marker effects and variances fluctuate across population subsets and highlight instabilities that may affect GWAS interpretation. For assessing robustness over time, it is useful to jointly evaluate GEBV persistence and SNP effect stability: while GEBV correlations inform predictive utility and deployment across generations, SNP effect reproducibility is critical for downstream fine-mapping and biological interpretation of candidate loci. Recency of training data is critical, as using more recent generations improves LD consistency and prediction performance. Polygenic traits are more robust to changes in training data. Major QTL signals are stable when the dataset is reduced, but small-effect background signals require larger and more connected training populations. Still, different subsamples resulted in false-positive and false-negative GWAS peaks, even when the prediction did not differ. That may result in unstable indirect predictions when young animals’ EBVs are calculated based on SNP effects [73]. Declines in indirect prediction accuracy were observed when the number of animals in genomic recursions was inappropriate, and decreased accuracy can occur over time [74]. Together, these results emphasize that while genomic prediction and discovery depend on similar factors, the robustness of GWAS is more sensitive to sampling than the quality of predictions. Therefore, while the performance of genomic prediction models is less affected by subsampling, genomic regions discovered can vary more broadly, especially when the sample size is small. Similar issues could be faced in the implementation of weighted analysis using ssGBLUP [8], since the calculation of such weights may vary over time and produce unstable results. The limitations of this simulation include the chromosome number and size, the lack of selection, the population structure not reflecting common livestock data, and the absence of genotype-by-environment interactions. Such limitations were designed to narrow the findings to mathematical and statistical explanations for the greater variation in GWAS results compared to predictions. Real data studies may also show fluctuations due to population structure and biological reasons [75,76]. Future studies should address the impact of this variation using different core animals in genomic recursions [58] and with sample sizes that reach the higher accuracy plateau, as well as using different methods and priors. Additionally, the number of replicates used in this study may be insufficient to interpret GWAS results, particularly since visual inspection of Manhattan plots was performed only for the first simulation. Those concerns are diminished by the low SE across the quantitative comparison of GWAS across the five replicates.

## 5. Conclusions

This study evaluated how training subset design influences genomic prediction accuracy, GEBV stability, SNP-effect consistency, and GWAS signal detection using different genetic architectures. Across scenarios, performance was primarily driven by training population size, generational proximity, and the number of underlying QTL. Complete training sets yielded the highest accuracies, whereas data partitioning reduced accuracy due to smaller training populations and reduced genetic connectedness. However, these changes in accuracy were small. Recent generations produced higher accuracies when compared to generations that were distant from the validation population, reflecting stronger persistence of LD and realized relationships. Polygenic architectures with 1000 QTLs resulted in higher and robust accuracies than oligogenic architectures with 100 QTLs, while the effect distribution (normal vs. gamma) had comparatively minor influence. GEBV correlations were consistently higher than SNP-effect correlations, indicating that genomic predictions are more robust than individual marker estimates. GWAS analyses showed that some major QTL peaks were stable, but false positives and false-negative signals were more often observed in reduced scenarios than in the complete data. Additionally, intermediate GWAS signals varied more than major ones, particularly in reduced subsets, highlighting the impact of statistical power and subset structure on detectability. The results emphasize that genomic prediction models can yield stable results with minor fluctuations if the sample size is appropriate. However, association studies are more impacted by data subsetting, which may influence QTL discovery.

## Figures and Tables

**Table 1:** Mean (SE) top-ranked SNP windows overlapping across subsets in different genetic architectures in TR_ID scenario.

Genetic Architecture	TR_ID Subset Comparison	Top 10	Top 100	Top 200
N-100	*All* vs. *Odd*	5.2 (0.37)	46.4 (2.10)	102.6 (4.65)
N-100	*Odd* vs. *Even*	2.1 (0.24)	25.8 (1.52)	61.4 (3.20)
N-100	*All* vs. *Even*	5.4 (0.40)	48.2 (2.18)	105.1 (4.82)
N-1000	*All* vs. *Odd*	7.1 (0.33)	58.6 (2.35)	121.7 (5.05)
N-1000	*Odd* vs. *Even*	3.2 (0.30)	34.5 (1.80)	76.2 (3.75)
N-1000	*All* vs. *Even*	7.8 (0.29)	61.3 (2.42)	126.4 (5.18)
G-100	*All* vs. *Odd*	4.3 (0.32)	42.1 (1.95)	94.3 (4.30)
G-100	*Odd* vs. *Even*	2.2 (0.22)	22.6 (1.35)	55.7 (3.05)
G-100	*All* vs. *Even*	5.0 (0.36)	45.3 (2.05)	99.4 (4.52)
G-1000	*All* vs. *Odd*	6.2 (0.35)	55.4 (2.28)	118.5 (4.95)
G-1000	*Odd* vs. *Even*	4.1 (0.31)	37.2 (1.88)	81.3 (3.90)
G-1000	*All* vs. *Even*	7.0 (0.32)	59.2 (1.16)	124.2 (1.77)

**Table 2:** Summary of high-variance SNP windows and QTL detection across TR_ID animal-ID subsets across genetic architectures (Mean (SE)).

Genetic Architecture	TR_ID Subset	QTL Threshold	Total Windows Above Threshold	TP Windows	FP Windows
N-100	*All*	0.20	5.2 (0.37)	4.1 (0.24)	1.1 (0.22)
N-100	*Odd*	0.20	5.0 (0.32)	3.1 (0.29)	1.9 (0.25)
N-100	*Even*	0.20	5.1 (0.34)	3.2 (0.28)	1.9 (0.24)
N-1000	*All*	0.10	8.4 (0.51)	6.2 (0.37)	2.2 (0.33)
N-1000	*Odd*	0.10	7.8 (0.44)	5.3 (0.34)	2.5 (0.29)
N-1000	*Even*	0.10	8.1 (0.48)	5.6 (0.36)	2.5 (0.31)
G-100	*All*	0.30	6.1 (0.40)	5.0 (0.32)	1.1 (0.22)
G-100	*Odd*	0.30	5.9 (0.36)	4.1 (0.31)	1.8 (0.24)
G-100	*Even*	0.30	6.0 (0.38)	4.0 (0.30)	2.0 (0.26)
G-1000	*All*	0.10	9.0 (0.55)	6.7 (0.42)	2.3 (0.35)
G-1000	*Odd*	0.10	8.5 (0.49)	5.8 (0.39)	2.7 (0.32)
G-1000	*Even*	0.10	8.7 (0.52)	6.0 (0.40)	2.7 (0.34)

For the G-1000 and N-1000 scenarios, only the top 100 simulated QTLs were investigated.

## Data Availability

No publicly archived datasets were analyzed in this study. All data were simulated, and the R script used to generate the simulated data and implement the simulation design (simulation_design.R) is provided with the submission.

## Supplementary Materials

The following supporting information can be downloaded at: https://www.mdpi.com/article/10.3390/genes17060670/s1, Figure S1: Manhattan plots for TR_Gen under N-1000; Figure S2: Manhattan plots for TR_Gen under N-100; Figure S3: Manhattan plots for TR_GenBlock under N-1000; Figure S4: Manhattan plots for TR_GenBlock under N-100; Figure S5: Manhattan plots for TR_ID under N-1000; Figure S6: Manhattan plots for TR_ID under N-100; Figure S7: Manhattan plots for TR_Sex under N-1000; Figure S8: Manhattan plots for TR_Sex under N-100; Figure S9: Manhattan plots for TR_Gen under G-1000; Figure S10: Manhattan plots for TR_Gen under G-100; Figure S11: Manhattan plots for TR_Sex under G-1000; Figure S12: Manhattan plots for TR_Sex under G-100; Table S1: Prediction accuracy and inter-subset GEBV and SNP effect correlations for TR_ID across four simulated genetic architectures; Table S2: Prediction accuracy and inter-subset GEBV and SNP effect correlations for TR_Gen across four simulated genetic architectures; Table S3: Prediction accuracy and inter-subset GEBV and SNP effect correlations for TR_GenBlock across four simulated genetic architectures; Table S4: Prediction accuracy and inter-subset GEBV and SNP effect correlations between all and generation blocks in TR_GenBlock across four simulated genetic architectures; Table S5: Prediction accuracy and inter-subset GEBV and SNP effect correlations for TR_Sex across four simulated genetic architectures.

## Abbreviations

The following abbreviations are used in this manuscript:

GWAS	Genome-wide association study
GEBV	Genomic estimated breeding value
GRM	Genomic Relationship Matrix
SNP	Single-nucleotide polymorphism
QTL	Quantitative trait loci
TBV	True breeding value
LD	Linkage disequilibrium
ssGBLUP	Single-step genomic best linear unbiased prediction
GBLUP	Genomic best linear unbiased prediction
TR_ID	Training scenario based on animal identification number
TR_Gen	Training scenario based on generation subset
TR_GenBlock	Training scenario based on generation blocks
TR_Sex	Training scenario based on sex
N-100	Normal distribution with 100 QTL
G-100	Gamma distribution with 100 QTL
N-1000	Normal distribution with 1000 QTL
G-1000	Gamma distribution with 1000 QTL
USDA-NIFA	United States Department of Agriculture, National Institute of Food and Agriculture
