# Supplementary material for: Effect of Genetic Architecture and Partitioning of Training Population on GEBVs, SNP Effects and GWAS: A Simulation Study
Source: Genes (Basel). 2026 Jun 7;17(6):670. doi: 10.3390/genes17060670 (PMC13299996; doi:10.3390/genes17060670)
Supplement: Supplementary file 1 [file genes-17-00670-s001.zip › genes-4292220-supplementary.pdf]

genes MDPI

Supplementary Table S1. Prediction accuracy and inter-subset stability for the animal IDs configuration (TR_ID) across four simulated genetic architectures. Values are presented as mean (SE) across five replicates. Accuracy is expressed as the Pearson correlation between true breeding values (TBV) and genomic estimated breeding values (GEBV). Inter-subset stability is represented by the correlations of GEBVs and SNP effects between the *Odd* and *Even* partitions. Genetic architectures are denoted by effect distribution (N: Normal; G: Gamma) and total QTL density (100 or 1,000 causative loci).

**Scenario TR_ID**									
**Genetic Architecture**	**Prediction Accuracy**			**Correlation between GEBVs**			**Correlation between SNP effects**		
	All	Odd	Even	All vs Odd	Odd vs Even	All vs Even	All vs Odd	Odd vs Even	All vs Even
**N-1000**	0.54 (0.003) aA	0.45 (0.002) aC	0.46 (0.002) bB	0.83 (0.006) aA	0.40 (0.010) aB	0.81 (0.006) aA	0.75 (0.008) aA	0.18 (0.012) bB	0.75 (0.008) aA
**G-1000**	0.55 (0.003) aA	0.45 (0.002) aC	0.47 (0.002) aB	0.84 (0.006) aA	0.38 (0.011) aC	0.79 (0.007) aB	0.76 (0.008) aA	0.20 (0.012) bB	0.75 (0.008) aA
**N-100**	0.47 (0.003) bA	0.37 (0.002) bC	0.38 (0.002) cB	0.79 (0.007) bA	0.31 (0.011) bB	0.79 (0.007) aA	0.76 (0.008) aA	0.22 (0.011) abB	0.75 (0.008) aA
**G-100**	0.48 (0.003) bA	0.37 (0.002) bC	0.38 (0.002) cB	0.78 (0.007) bA	0.30 (0.011) bB	0.79 (0.007) aA	0.75 (0.008) aA	0.25 (0.011) aB	0.75 (0.008) aA

Table note. Values are presented as mean (SE). Lowercase letters compare genetic architectures within the same column. Uppercase letters compare generation blocks or pairwise scenario comparisons within the same genetic architecture and metric. Means sharing the same letter are not significantly different based on Tukey's HSD test. GEBV = genomic estimated breeding value.

Supplementary Table S2. Prediction accuracy and inter-subset stability for the alternating generation configuration (TR_Gen) across four simulated genetic architectures. Values are presented as mean (SE) across five replicates. Accuracy is expressed as the Pearson correlation between true breeding values (TBV) and genomic estimated breeding values (GEBV). Inter-subset stability is represented by the correlations of GEBVs and SNP effects between the *Odd_gen* and *Even_gen* partitions. Genetic architectures are denoted by effect distribution (N: Normal; G: Gamma) and total QTL density (100 or 1,000 causative loci).

**Scenario TR_Gen**									
**Genetic Architecture**	**Prediction Accuracy**			**Correlation between GEBVs**			**Correlation between SNP effects**		
	All	Odd_gen	Even_gen	All vs Odd_gen	Odd vs Even_gen	All vs Even_gen	All vs Odd_gen	Odd_gen vs Even_gen	All vs Even_gen
N-1000	0.54 (0.002) aA	0.44 (0.002) aC	0.48 (0.005) aB	0.71 (0.006) aB	0.45 (0.005) aC	0.75 (0.008) aA	0.75 (0.007) aA	0.19 (0.003) cC	0.71 (0.007) aB

G-1000	0.55 (0.004) aA	0.45 (0.003) aC	0.47 (0.006) aB	0.70 (0.006) abB	0.43 (0.006) bC	0.73 (0.008) aA	0.76 (0.006) aA	0.22 (0.002) bC	0.71 (0.006) aB
N-100	0.47 (0.002) bA	0.36 (0.008) bB	0.37 (0.007) bB	0.68 (0.007) bcA	0.31 (0.005) cB	0.67 (0.009) bA	0.75 (0.009) aA	0.23 (0.005) bC	0.70 (0.005) aB
G-100	0.48 (0.005) bA	0.36 (0.006) bB	0.37 (0.005) bB	0.67 (0.007) cA	0.30 (0.001) cB	0.67 (0.009) bA	0.76 (0.005) aA	0.26 (0.005) aC	0.70 (0.009) aB

Table note. Values are presented as mean (SE). Lowercase letters compare genetic architectures within the same column. Uppercase letters compare generation blocks or pairwise scenario comparisons within the same genetic architecture and metric. Means sharing the same letter are not significantly different based on Tukey's HSD test. GEBV = genomic estimated breeding value.

Supplementary Table S3 Prediction accuracy and inter-subset stability for within generation blocks configuration (TR_GenBlock) across four simulated genetic architectures. Values are presented as mean (SE) across five replicates. Accuracy is expressed as the Pearson correlation between true breeding values (TBV) and genomic estimated breeding values (GEBV). Inter-subset stability is represented by the correlations of GEBVs and SNP effects between the *Gen1-3, Gen4-6* and *Gen7-9* partitions. Genetic architectures are denoted by effect distribution (N: Normal; G: Gamma) and total QTL density (100 or 1,000 causative loci).

**Scenario TR_Gen (Within Generation Blocks)**										
**Genetic Architecture**		**Prediction Accuracy**			**GEBV**			**SNP effects**		
	All	Gen 1_3	Gen 4_6	Gen 7_9	Gen 1_3 vs Gen 4_6	Gen 4_6 vs Gen 7_9	Gen 7_9 vs Gen 1_3	Gen 1_3 vs Gen 4_6	Gen 4_6 vs Gen 7_9	Gen 7_9 vs Gen 1_9
N-1000	0.54 (0.002) aA	0.44 (0.002) aC	0.48 (0.005) aB	0.71 (0.006) aB	0.45 (0.005) aC	0.75 (0.008) aA	0.75 (0.007) aA	0.19 (0.003) cC	0.71 (0.007) aB	0.54 (0.002) aA
G-1000	0.55 (0.004) aA	0.45 (0.003) aC	0.47 (0.006) aB	0.70 (0.006) abB	0.43 (0.006) bC	0.73 (0.008) aA	0.76 (0.006) aA	0.22 (0.002) bC	0.71 (0.006) aB	0.55 (0.004) aA
N-100	0.47 (0.002) bA	0.36 (0.008) bB	0.37 (0.007) bB	0.68 (0.007) bcA	0.31 (0.005) cB	0.67 (0.009) bA	0.75 (0.009) aA	0.23 (0.005) bC	0.70 (0.005) aB	0.47 (0.002) bA
G-100	0.48 (0.005) bA	0.36 (0.006) bB	0.37 (0.005) bB	0.67 (0.007) cA	0.30 (0.001) cB	0.67 (0.009) bA	0.76 (0.005) aA	0.26 (0.005) aC	0.70 (0.009) aB	0.48 (0.005) bA

Table note. Values are presented as mean (SE). Lowercase letters compare genetic architectures within the same column. Uppercase letters compare generation blocks or pairwise scenario comparisons within the same genetic architecture and metric. Means sharing the same letter are not significantly different based on Tukey's HSD test. GEBV = genomic estimated breeding value.

Supplementary Table S4. Prediction accuracy and inter-subset stability between all vs generation blocks configuration (TR_GenBlock) across four simulated genetic architectures. Accuracy is expressed as the Pearson correlation between true breeding values (TBV) and genomic estimated breeding values (GEBV). Inter-subset stability is represented by the correlations of GEBVs and SNP effects between the *Gen 1_3, Gen 4_6* and *Gen 7_9* partitions. Genetic architectures are denoted by effect distribution (N: Normal; G: Gamma) and total QTL density (100 or 1,000 causative loci).

**Scenario TR_Gen (All vs Generation Blocks comparisons)**										
**Genetic Architecture**		**Prediction Accuracy**			**GEBV**			**SNP effects**		
	All	Gen 1_3	Gen 4_6	Gen 7_9	All vs Gen 1_3	All vs Gen 4_6	All vs Gen 7_9	All vs Gen 1_3	All vs Gen 4_6	All vs Gen 1_3
N-1000	0.54 (0.002) aA	0.44 (0.002) aC	0.48 (0.005) aB	0.71 (0.006) aB	0.45 (0.005) aC	0.75 (0.008) aA	0.75 (0.007) aA	0.19 (0.003) cC	0.71 (0.007) aB	0.54 (0.002) aA
G-1000	0.55 (0.004) aA	0.45 (0.003) aC	0.47 (0.006) aB	0.70 (0.006) abB	0.43 (0.006) bC	0.73 (0.008) aA	0.76 (0.006) aA	0.22 (0.002) bC	0.71 (0.006) aB	0.55 (0.004) aA
N-100	0.47 (0.002) bA	0.36 (0.008) bB	0.37 (0.007) bB	0.68 (0.007) bcA	0.31 (0.005) cB	0.67 (0.009) bA	0.75 (0.009) aA	0.23 (0.005) bC	0.70 (0.005) aB	0.47 (0.002) bA
G-100	0.48 (0.005) bA	0.36 (0.006) bB	0.37 (0.005) bB	0.67 (0.007) cA	0.30 (0.001) cB	0.67 (0.009) bA	0.76 (0.005) aA	0.26 (0.005) aC	0.70 (0.009) aB	0.48 (0.005) bA

Table note. Values are presented as mean (SE). Lowercase letters compare genetic architectures within the same column. Uppercase letters compare generation blocks or pairwise scenario comparisons within the same genetic architecture and metric. Means sharing the same letter are not significantly different based on Tukey's HSD test. GEBV = genomic estimated breeding value.

Supplementary Table S5. Prediction accuracy and inter-subset stability for sex configuration (TR_Sex) across four simulated genetic architectures. Values are presented as mean (SE) across five replicates. Accuracy is expressed as the Pearson correlation between true breeding values (TBV) and genomic estimated breeding values (GEBV). Inter-subset stability is represented by the correlations of GEBVs and SNP effects between the *Male and Female* partitions. Genetic architectures are denoted by effect distribution (N: Normal; G: Gamma) and total QTL density (100 or 1,000 causative loci).

**Scenario TR_Sex**									
**Genetic Architecture**	**Prediction Accuracy**			**Correlation between GEBVs**			**Correlation between SNP effects**		
	All	Male	Female	All vs Male	All vs Female	Male vs Female	All vs Male	All vs Female	Male vs Female
**N-1000**	0.54 (0.003) aA	0.45 (0.005) aB	0.46 (0.008) aB	0.80 (0.004) bA	0.78 (0.002) aB	0.41 (0.004) aC	0.78 (0.004) aA	0.75 (0.002) bB	0.21 (0.003) cC
**G-1000**	0.55 (0.004) aA	0.44 (0.006) aB	0.45 (0.009) aB	0.84 (0.005) aA	0.79 (0.003) aB	0.40 (0.005) aC	0.79 (0.007) aA	0.76 (0.003) bB	0.22 (0.002) bC

**N-100**	0.47 (0.003) bA	0.37 (0.005) bB	0.36 (0.008) bB	0.78 (0.006) bA	0.76 (0.004) bB	0.38 (0.003) bC	0.76 (0.006) bA	0.77 (0.004) aA	0.23 (0.004) bB
**G-100**	0.48 (0.002) bA	0.38 (0.004) bB	0.38 (0.003) bB	0.79 (0.005) bA	0.76 (0.001) bB	0.39 (0.001) bC	0.77 (0.007) aA	0.78 (0.005) aA	0.25 (0.004) aB

Table note. Values are presented as mean (SE). Lowercase letters compare genetic architectures within the same column. Uppercase letters compare generation blocks or pairwise scenario comparisons within the same genetic architecture and metric. Means sharing the same letter are not significantly different based on Tukey's HSD test. GEBV = genomic estimated breeding value.

Figure S1. Manhattan plots for the N-1000 genetic architecture across TR_Gen subsets (All, Odd_gen, and Even_gen) based on generation number. The left y-axis represents SNP-window variance explained; the right y-axis represents the proportion of QTL variance (%). Red markers indicate true simulated QTL positions. Alternating colors distinguish ten chromosomes across the genome.

Figure S2. Manhattan plots for the N-100 genetic architecture across TR_Gen subsets (All, Odd_gen, and Even_gen) based on generation number. The left y-axis represents SNP-window variance explained; the right y-axis represents the proportion of QTL variance (%). Red markers indicate true simulated QTL positions. Alternating colors distinguish ten chromosomes across the genome.

Figure S3. Manhattan plots for the N-1000 genetic architecture across three generation blocks (Gen 1_3, Gen 4_6, and Gen 7_9) under the TR_GenBlock scenario. The left y-axis represents SNP-window variance explained; the right y-axis represents the proportion of QTL variance (%). Alternating colors distinguish ten chromosomes across the genome.

Figure S4. Manhattan plots for the N-100 genetic architecture across three generation blocks (Gen 1_3, Gen 4_6, and Gen 7_9) under the TR_GenBlock scenario. The left y-axis represents SNP-window variance explained; the right y-axis represents the proportion of QTL variance (%). Alternating colors distinguish ten chromosomes across the genome.

Figure S5. Manhattan plots for the N-1000 genetic architecture across TR_ID subsets (All, Odd, and Even) based on animal identification numbers. The left y-axis represents the proportion of genetic variance explained by individual SNPs; the right y-axis represents the proportion of QTL variance (%). Red markers indicate true simulated QTL positions. Alternating colors distinguish ten chromosomes across the genome.

Figure S6. Manhattan plots for the N-100 genetic architecture across TR_ID subsets (All, Odd, and Even) based on animal identification numbers. The left y-axis represents the proportion of genetic variance explained by individual SNPs; the right y-axis represents the proportion of QTL variance (%). Red markers indicate true simulated QTL positions. Alternating colors distinguish ten chromosomes across the genome.

Figure S7. Manhattan plots for the N-1000 genetic architecture across TR_Sex subsets (All, Male, and Female) based on sex classification. The left y-axis represents SNP-window variance explained; the right y-axis represents the proportion of QTL variance (%). Red markers indicate true simulated QTL positions. Alternating colors distinguish ten chromosomes across the genome.

Figure S8. Manhattan plots for the N-100 genetic architecture across TR_Sex subsets (All, Male, and Female) based on sex classification. The left y-axis represents SNP-window variance explained; the right y-axis represents the proportion of QTL variance (%). Red markers indicate true simulated QTL positions. Alternating colors distinguish ten chromosomes across the genome.

Figure S9. Manhattan plots for the G-1000 genetic architecture across TR_Gen subsets (All, Odd_gen, and Even_gen) based on generation number. The left y-axis represents SNP-window variance explained; the right y-axis represents the proportion of QTL variance (%). Red markers indicate true simulated QTL positions. Alternating colors distinguish ten chromosomes across the genome.

Figure S10. Manhattan plots for the G-100 genetic architecture across TR_Gen subsets (All, Odd_gen, and Even_gen) based on generation number. The left y-axis represents SNP-window variance explained; the right y-axis represents the proportion of QTL variance (%). Red markers indicate true simulated QTL positions. Alternating colors distinguish ten chromosomes across the genome.

Figure S11. Manhattan plots for the G-1000 genetic architecture across TR_Sex subsets (All, Male, and Female) based on sex classification. The left y-axis represents SNP-window variance explained; the right y-axis represents the proportion of QTL variance (%). Red markers indicate true simulated QTL positions. Alternating colors distinguish ten chromosomes across the genome.

Figure S12. Manhattan plots for the G-100 genetic architecture across TR_Sex subsets (All, Male, and Female) based on sex classification. The left y-axis represents SNP-window variance explained; the right y-axis represents the proportion of QTL variance (%). Red markers indicate true simulated QTL positions. Alternating colors distinguish ten chromosomes across the genome.

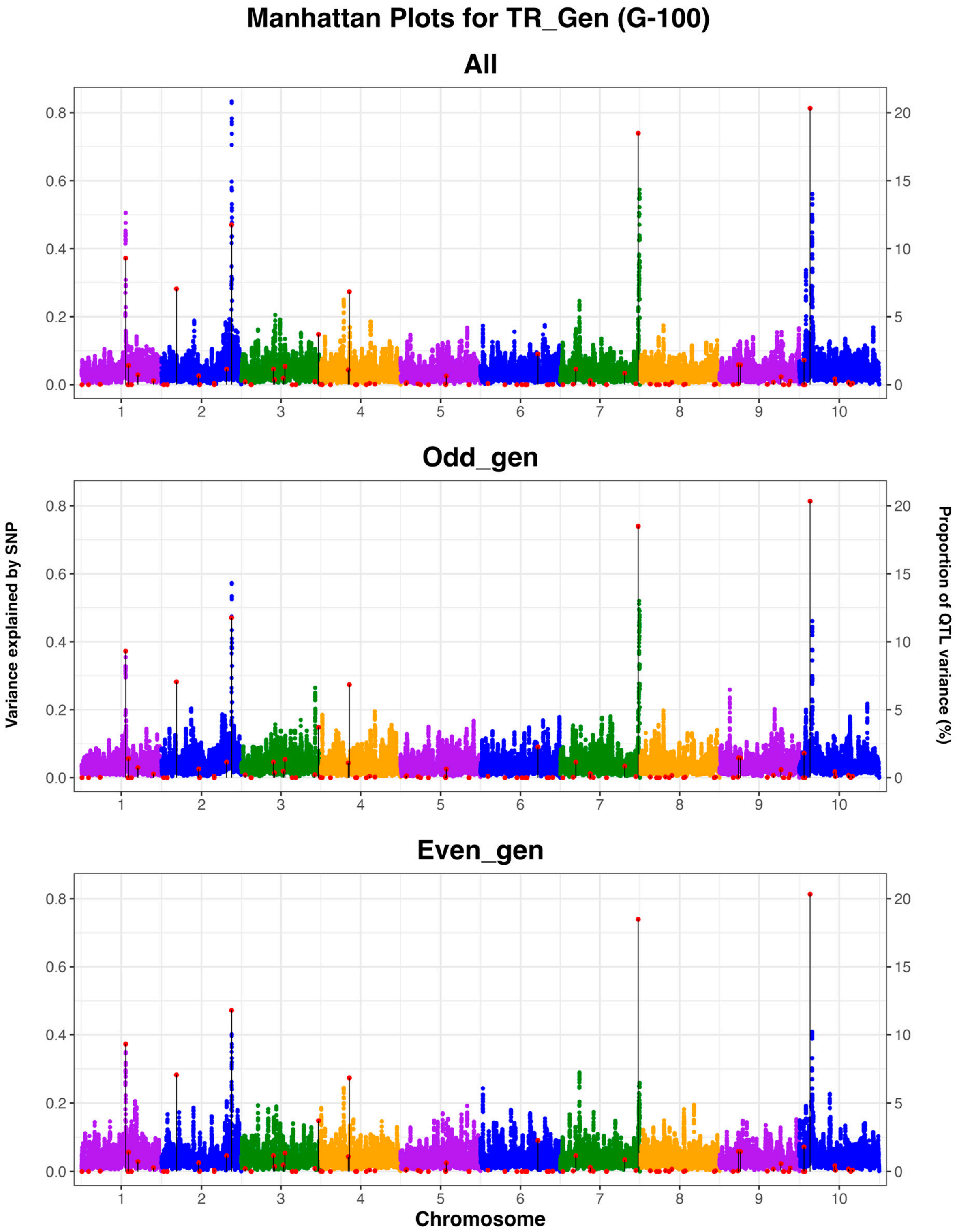
Manhattan Plots for TR_Gen (G-100)
All
Odd_gen
Even_gen
Variance explained by SNP
Proportion of QTL variance (%)
Chromosome
0.0
0.2
0.4
0.6
0.8
0
5
10
15
20
1 2 3 4 5 6 7 8 9 10

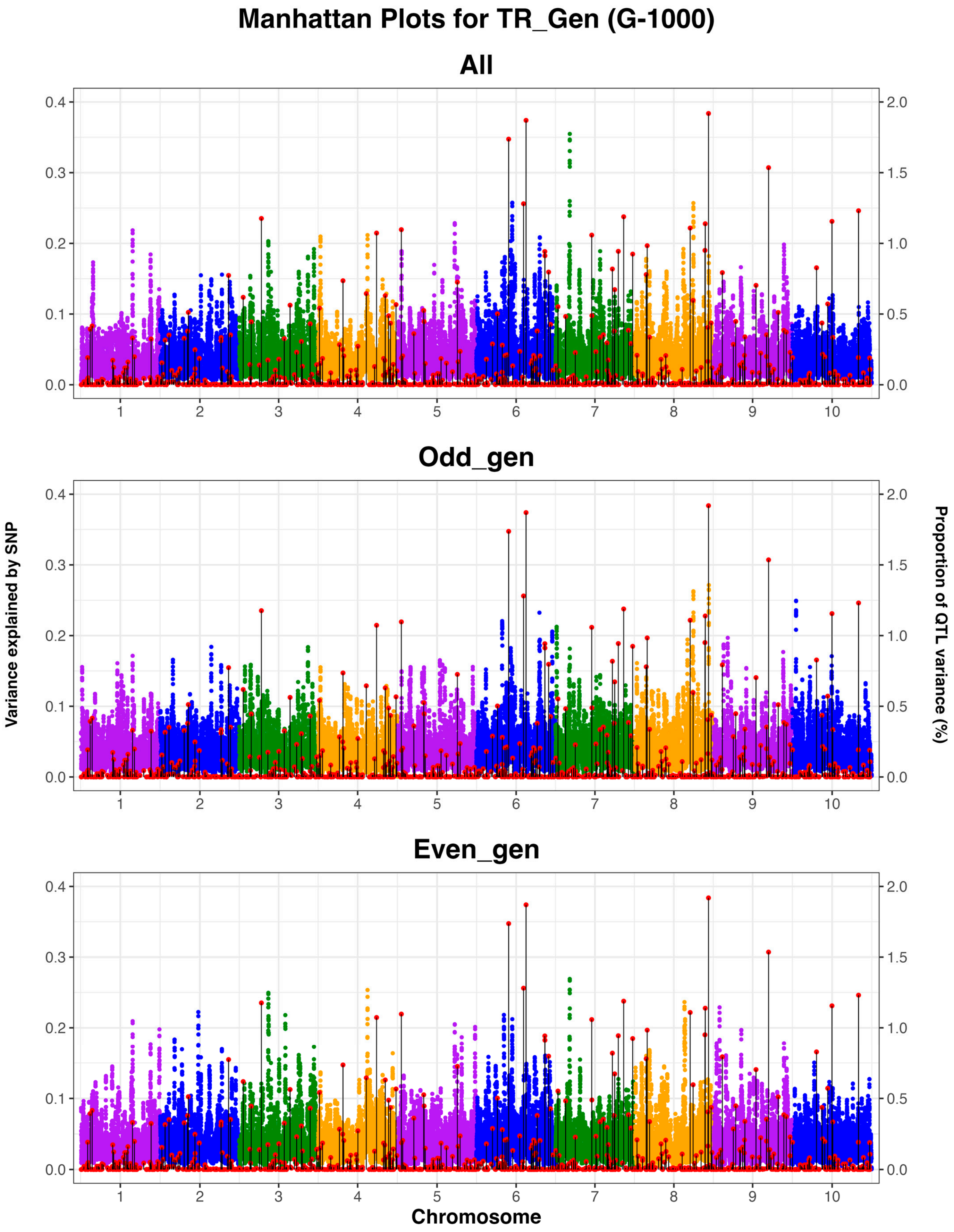

Manhattan Plots for TR_Gen (G-1000)
All
Odd_gen
Even_gen
Variance explained by SNP
Proportion of QTL variance (%)
Chromosome
0.0
0.1
0.2
0.3
0.4
0.5
1.0
1.5
2.0
1
2
3
4
5
6
7
8
9
10

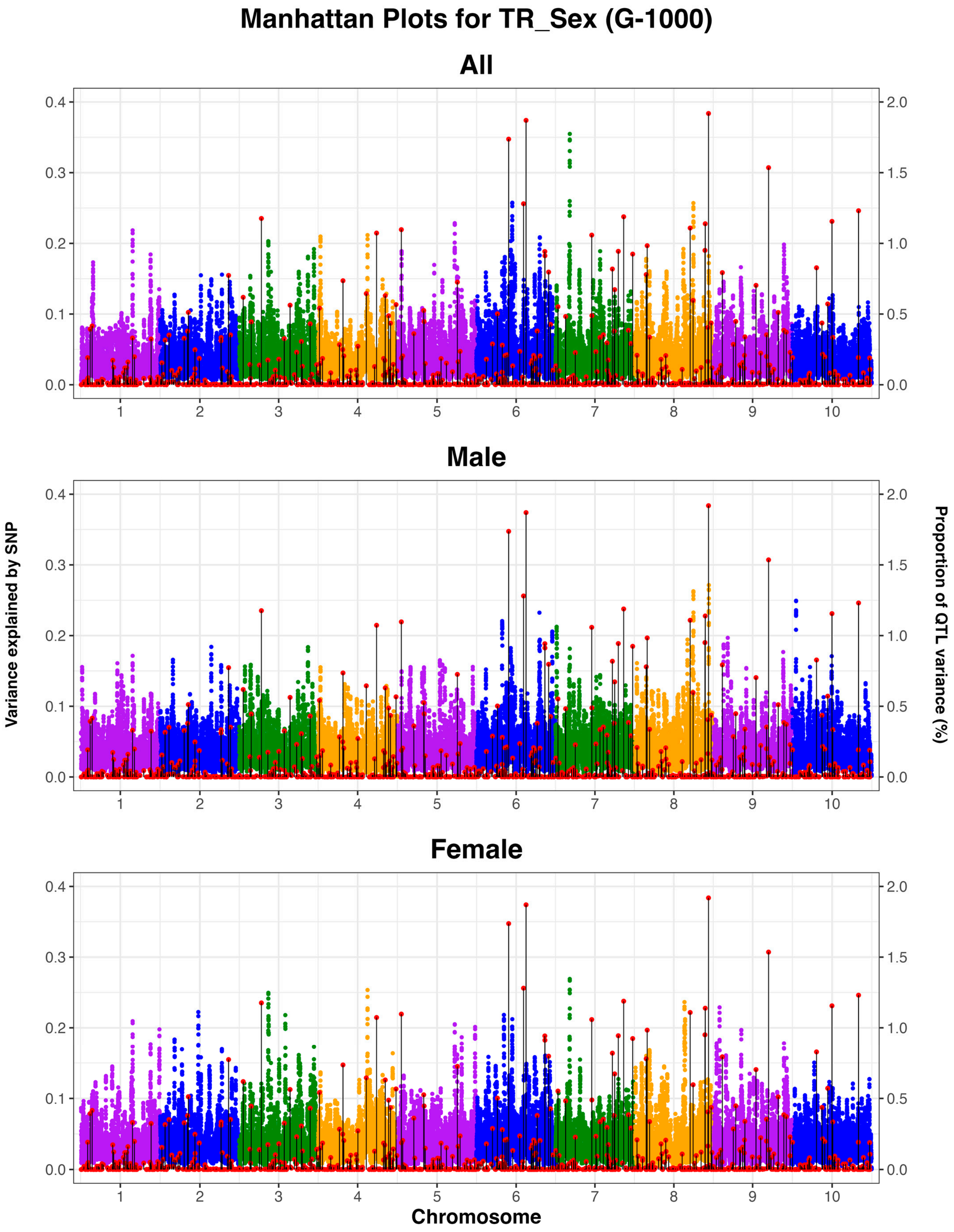
Manhattan Plots for TR_Sex (G-1000)
All
Male
Female
Variance explained by SNP
Proportion of QTL variance (%)
Chromosome
0.0
0.1
0.2
0.3
0.4
0.5
1.0
1.5
2.0
1
2
3
4
5
6
7
8
9
10

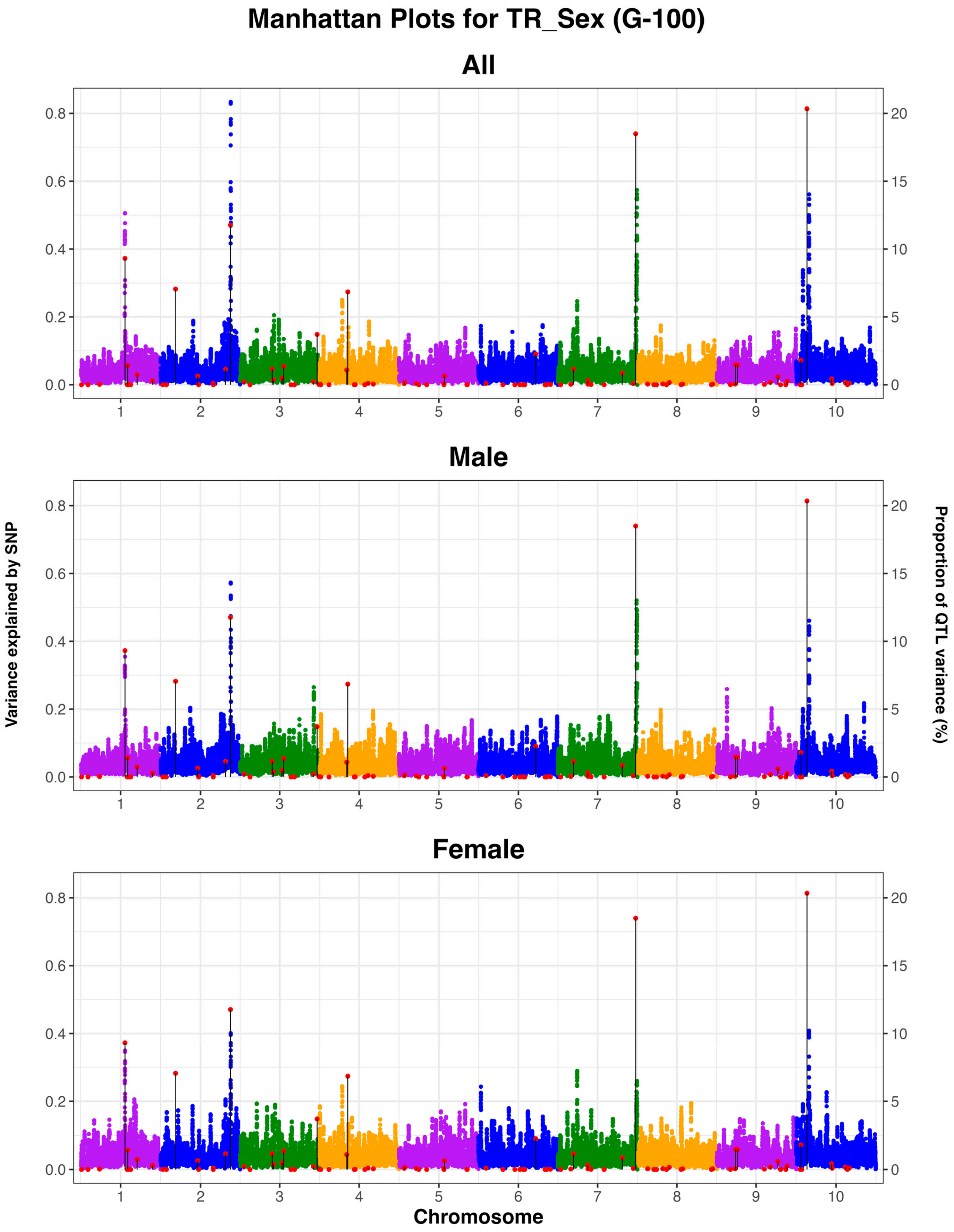
Manhattan Plots for TR_Sex (G-100)
All
Male
Female
Variance explained by SNP
Proportion of QTL variance (%)
Chromosome
0.0
0.2
0.4
0.6
0.8
0
5
10
15
20
1
2
3
4
5
6
7
8
9
10

Manhattan Plots

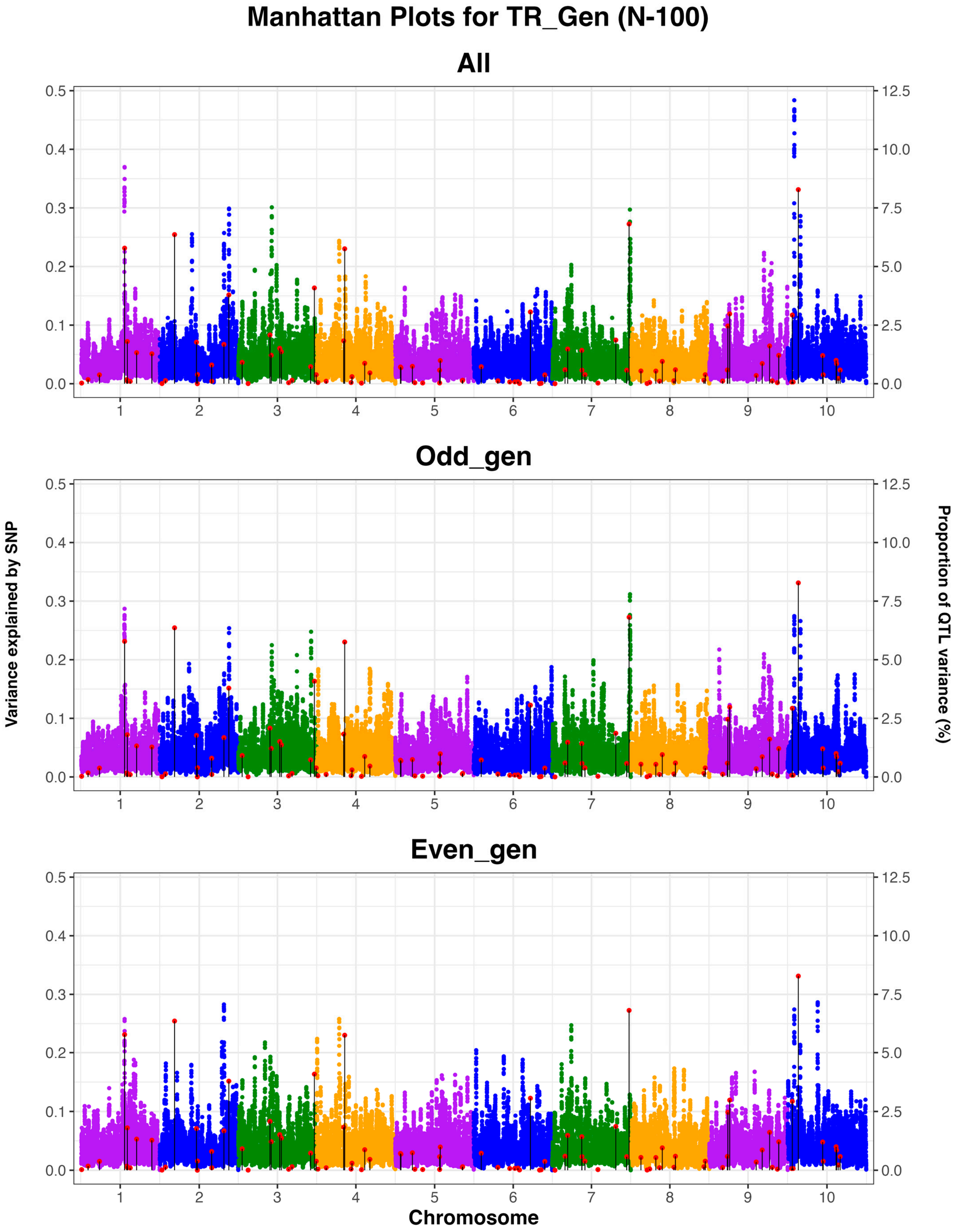

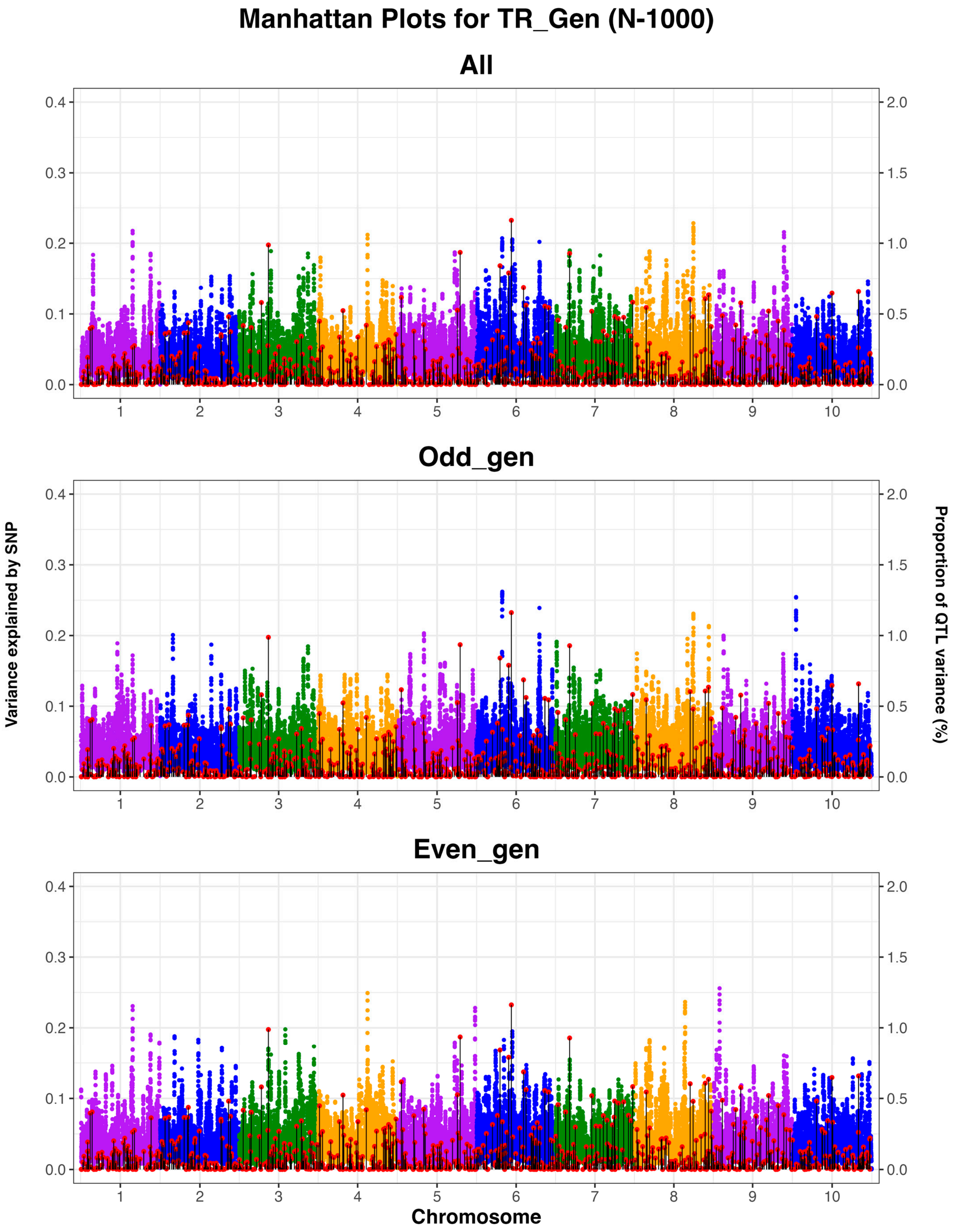
Manhattan Plots for TR_Gen (N-1000)
All
Odd_gen
Even_gen
Variance explained by SNP
Proportion of QTL variance (%)
Chromosome
0.0
0.1
0.2
0.3
0.4
0.5
1.0
1.5
2.0
1
2
3
4
5
6
7
8
9
10

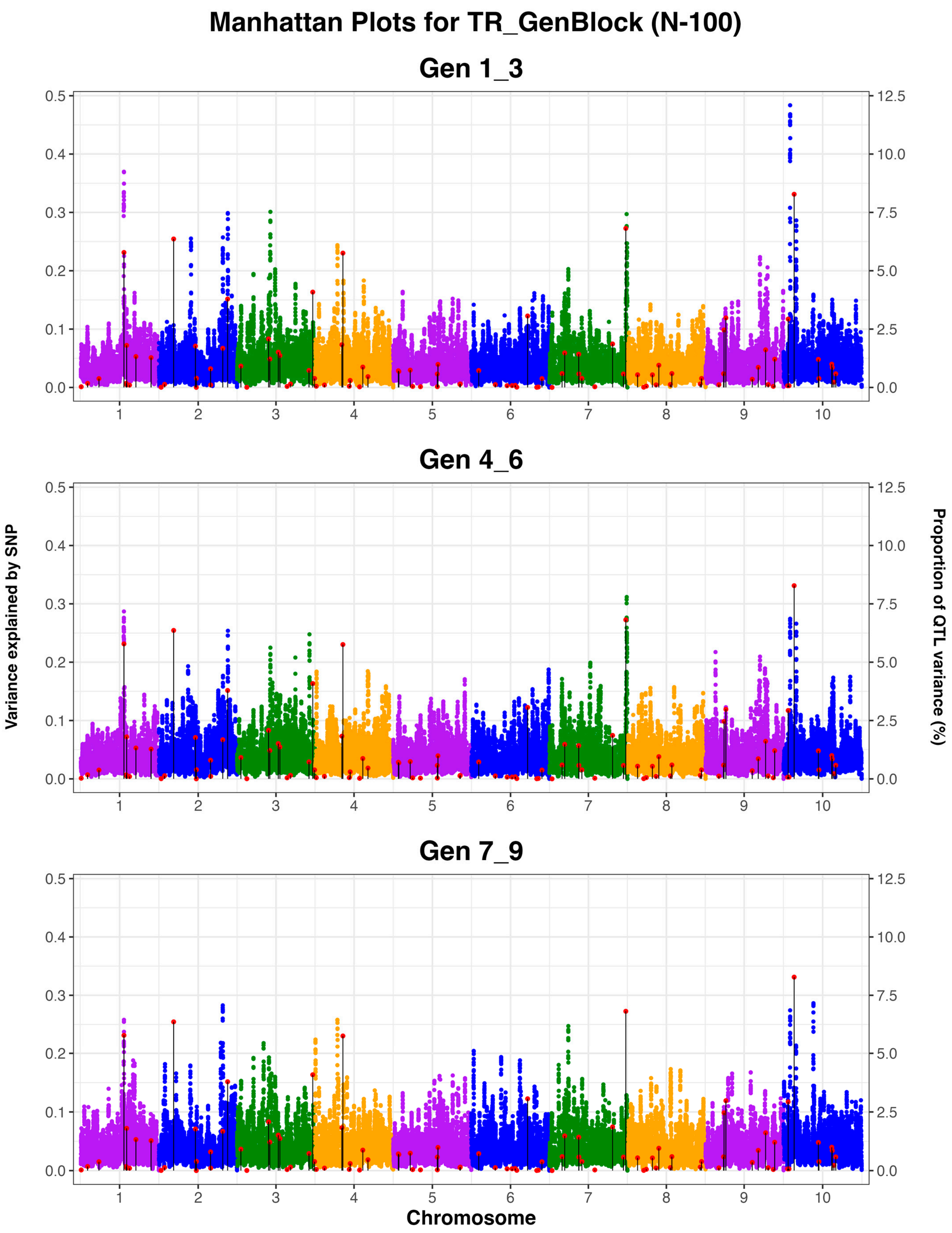
Manhattan Plots for TR_GenBlock (N-100)
Gen 1_3
Gen 4_6
Gen 7_9
Variance explained by SNP
Proportion of QTL variance (%)
Chromosome
0.0
0.1
0.2
0.3
0.4
0.5
0.0
2.5
5.0
7.5
10.0
12.5
1
2
3
4
5
6
7
8
9
10

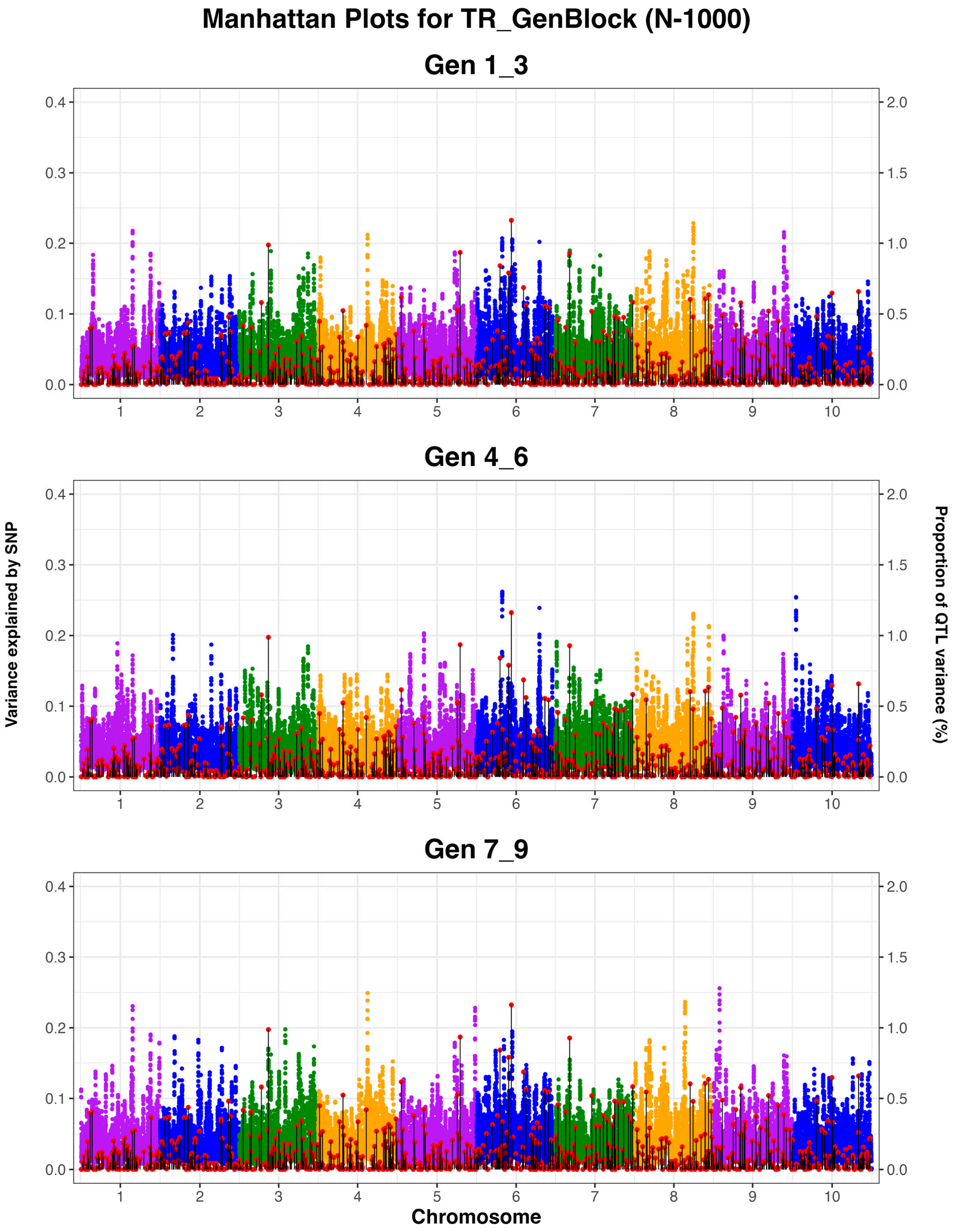
Manhattan Plots for TR_GenBlock (N-1000)
Gen 1_3
Gen 4_6
Gen 7_9
Variance explained by SNP
Proportion of QTL variance (%)
Chromosome
0.0
0.1
0.2
0.3
0.4
0.5
1.0
1.5
2.0
1
2
3
4
5
6
7
8
9
10

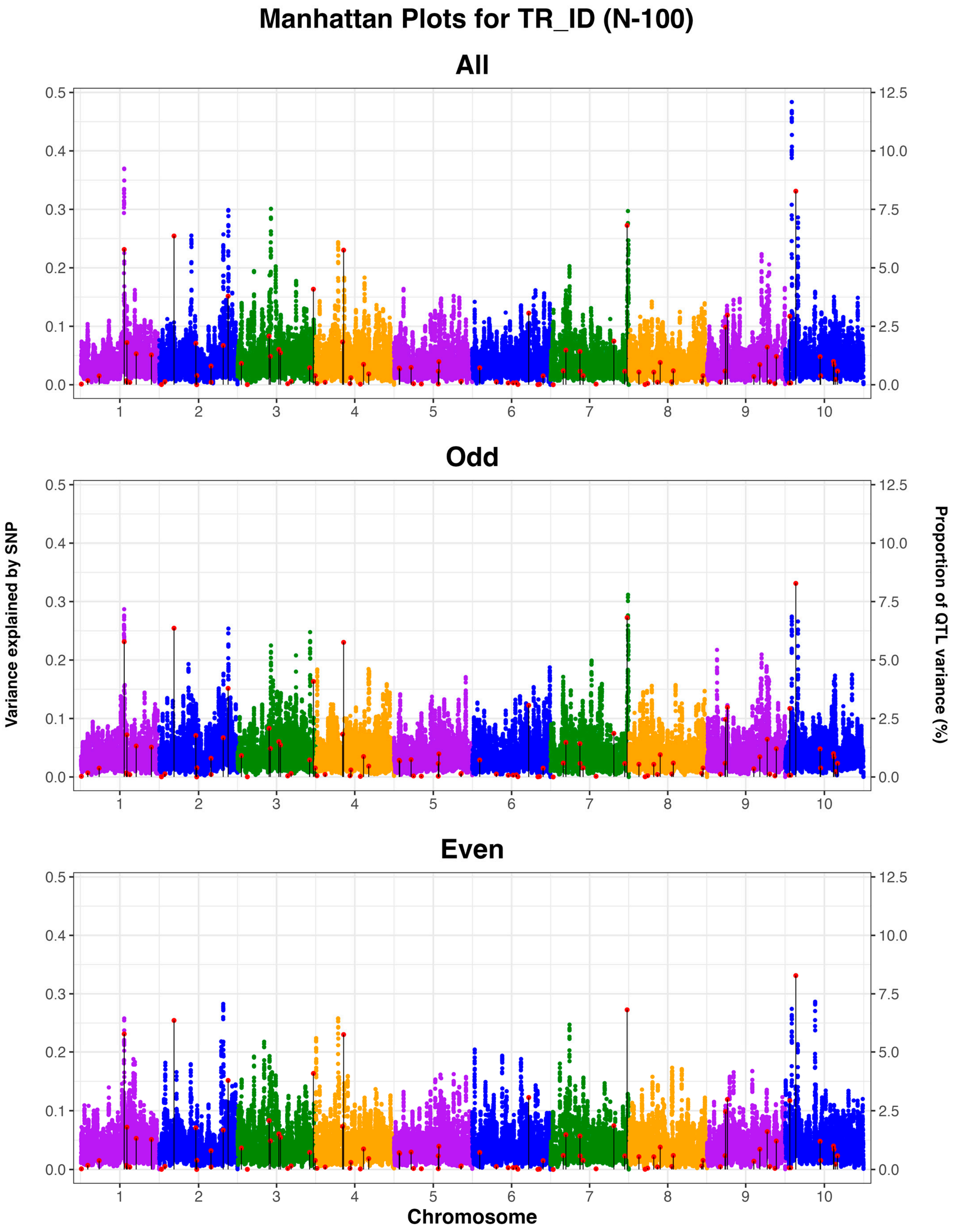
Manhattan Plots for TR_ID (N-100)
All
Odd
Even
Variance explained by SNP
Proportion of QTL variance (%)
Chromosome
0.0
0.1
0.2
0.3
0.4
0.5
0.0
2.5
5.0
7.5
10.0
12.5
1
2
3
4
5
6
7
8
9
10

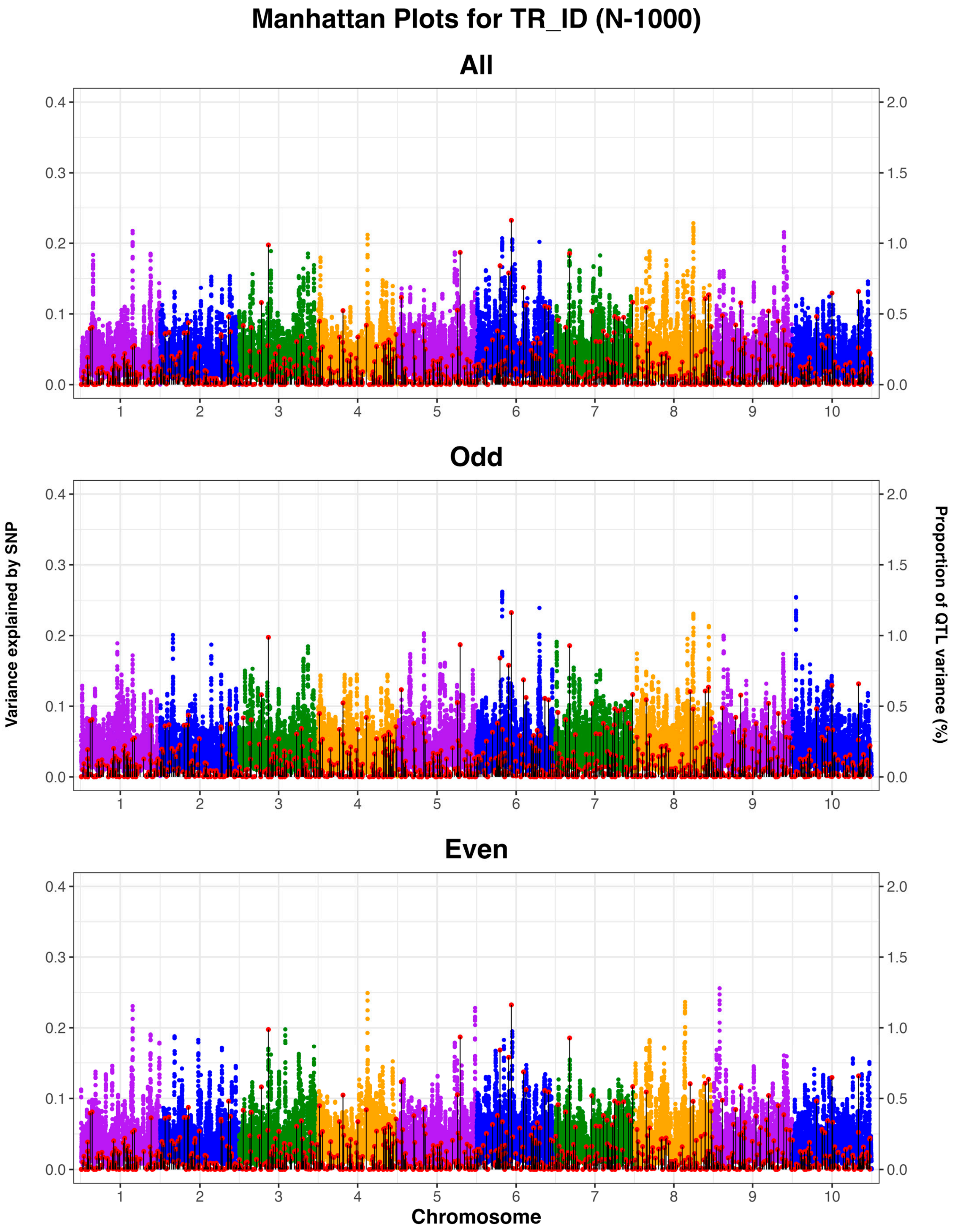
Manhattan Plots for TR_ID (N-1000)
All
Odd
Even
Variance explained by SNP
Proportion of QTL variance (%)
Chromosome
0.0
0.1
0.2
0.3
0.4
0.0
0.5
1.0
1.5
2.0
1
2
3
4
5
6
7
8
9
10

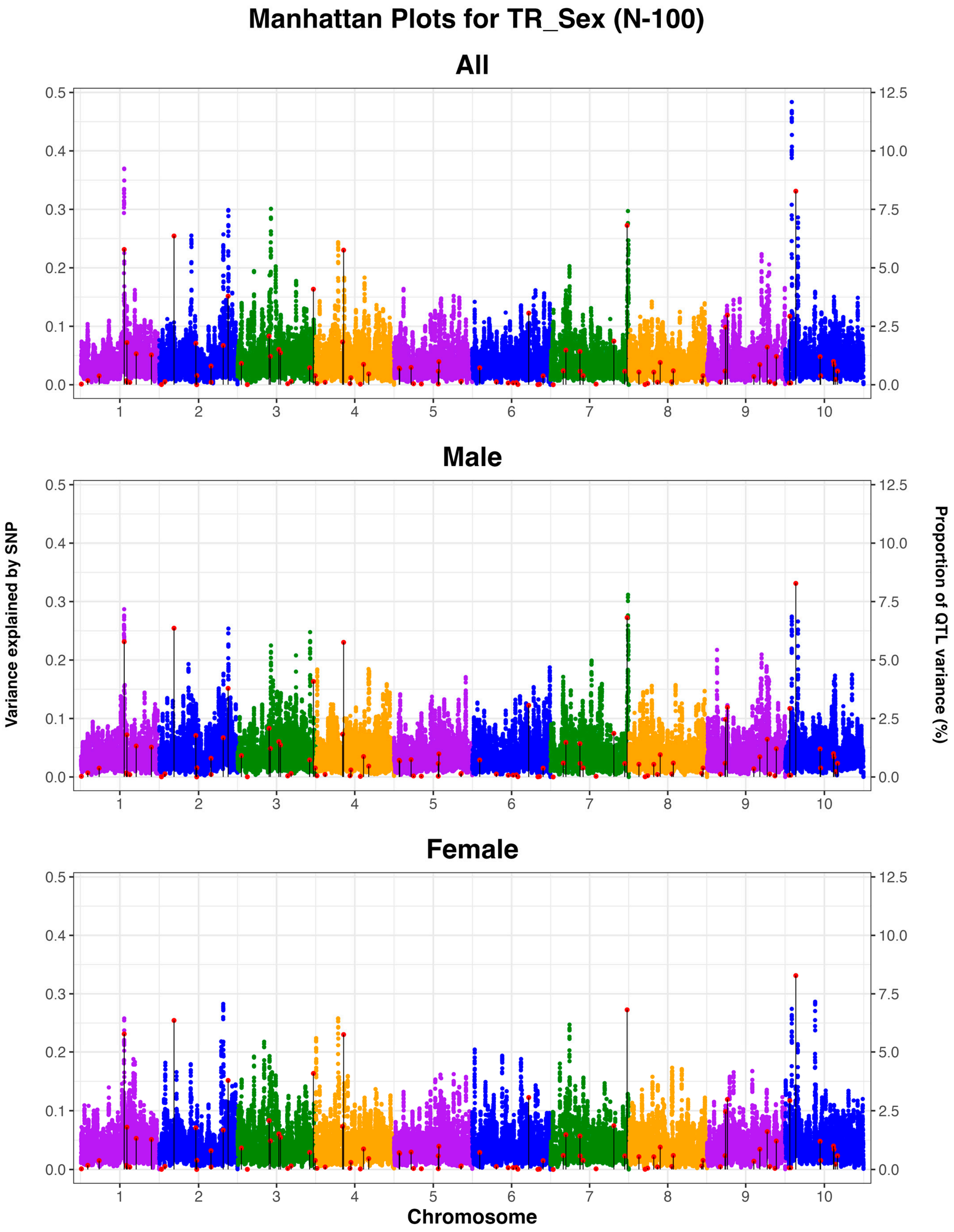
Manhattan Plots for TR_Sex (N-100)
All
Male
Female
Variance explained by SNP
Proportion of QTL variance (%)
Chromosome
0.0
0.1
0.2
0.3
0.4
0.5
0.0
2.5
5.0
7.5
10.0
12.5
1
2
3
4
5
6
7
8
9
10

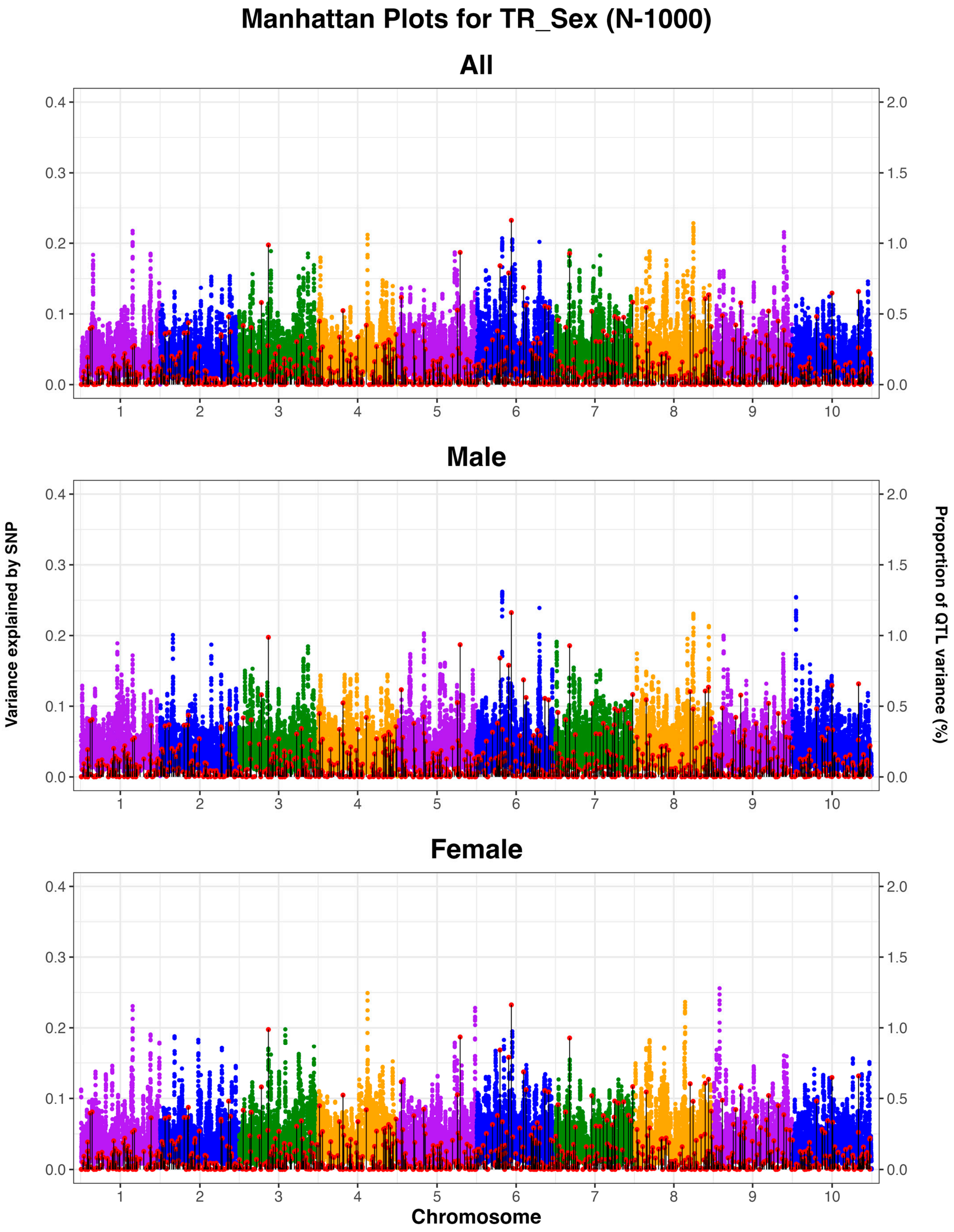
Manhattan Plots for TR_Sex (N-1000)
All
Male
Female
Variance explained by SNP
Proportion of QTL variance (%)
Chromosome
0.0
0.1
0.2
0.3
0.4
0.0
0.5
1.0
1.5
2.0
1
2
3
4
5
6
7
8
9
10
